# Exploring the Effects of Serious Games (Immersive Virtual Reality Versus Web-Based Platforms) on Interprofessional Education Among Undergraduate Health Care Students: Randomized Controlled Trial and Multimethod Study

**DOI:** 10.2196/80033

**Published:** 2026-05-25

**Authors:** Justina Yat Wa Liu, Rina Wing Yan Wong, Sabina Margaret Pinto, Curtis Ka Ho Wong, Patrick Pui Kin Kor, Shanshan Wang, Kitty Chan

**Affiliations:** 1 School of Nursing, Hong Kong Polytechnic University, 11 Yuk Choi Road, Hung Hom, Kowloon, Hong Kong, China (Hong Kong), 852 2766 4097; 2 Centre for Immersive Learning and Metaverse in Education, Hong Kong Polytechnic University, Hong Kong, China (Hong Kong); 3 School of Medical and Health Sciences, Tung Wah College, Hong Kong, China (Hong Kong); 4 Department of Rehabilitation Sciences, Hong Kong Polytechnic University, Hong Kong, China (Hong Kong)

**Keywords:** interprofessional education, virtual reality, undergraduate health care education, serious games, health care students, randomized controlled trial, educational technology

## Abstract

**Background:**

Interprofessional education (IPE) is essential for developing teamwork and communication skills in health care training. Building interprofessional competency involves acquiring knowledge, cultivating readiness to engage in teamwork, and fostering effective collaboration. However, challenges include geographical barriers and faculty resistance that can hinder progress. Innovative solutions to enhance engagement and collaboration include serious games played using immersive virtual reality (IVR) and web-based platforms. Despite their potential, research comparing the effectiveness of these approaches for IPE remains limited.

**Objective:**

This study aimed to identify effective implementation methods by evaluating the impact of IVR and web-based serious games on health care students’ interprofessional competencies and learning experiences.

**Methods:**

A multimethod approach was used to evaluate students’ pre- and postlearning outcomes and experiences. Nursing and physiotherapy students were randomly assigned to either the IVR group or web-based group. The Readiness for Interprofessional Learning Scale (RIPLS), Brief Sense of Community Scale (BSCS), and Intrinsic Motivation Inventory (IMI) were used to measure learning experiences. Learning outcomes were examined through multiple-choice questions (MCQs). After the outcome assessments, groups switched modalities to allow all students to experience both learning approaches. Four focus groups (n=34) provided qualitative insights into learning experiences.

**Results:**

The study included 271 students (IVR group: 125; web-based group: 146). Both groups showed significant improvements in community building (BSCS; IVR: *r*=−0.29, 95% CI 0.06-0.31; *P*=.001; web-based: *r*=−0.27, 95% CI 0.07-0.31; *P*=.001). The IVR group showed improvements in only the pressure/tension subscale of the IMI (*r*=−0.45, 95% CI −0.05 to −0.25; *P*<.001), while the web-based group showed improvements across all IMI subscales, including interest/enjoyment (*r***=**−0.17, 95% CI 0.00-0.30; *P*=.043), perceived competence (*r*=−0.19, 95% CI 0.00-0.50; *P*=.02), and pressure/tension (*r*=−0.46, 95% CI −0.75 to −0.50; *P*<.001). Both groups showed significant improvements in knowledge-checking MCQ scores (IVR: *r*=−0.64, 95% CI 2.00-3.00; *P*<.001; web-based: *r*=−0.80, 95% CI 4.50-6.00; *P*<.001), with the web-based group outperforming the IVR group (*r*=−0.34, 95% CI −3.00 to −2.00; *P*<.001). RIPLS scores showed no significant difference in both groups, suggesting that greater exposure is required to enhance IPE readiness. A qualitative analysis revealed 6 themes: valuing collaboration opportunities, fostering trust and shared learning, promoting engagement and authenticity, enhancing knowledge retention, balancing innovation with practicality, and improving realism and inclusivity. No adverse events were noted.

**Conclusions:**

To our knowledge, this is the first study to compare the effects of 2 different modalities (IVR and web-based approaches) on a technology-enabled IPE program. These findings highlight the practical potential of both approaches in supporting IPE. The web-based approach’s superior performance in enhancing learning outcomes and motivation suggests that its flexible format better accommodates diverse learning preferences. These findings support technology-enhanced IPE, offering educators scalable, evidence-based strategies to develop collaborative, patient-centered professionals who are equipped for real-world practice.

## Introduction

### Background and Rationale

Interprofessional education (IPE) is increasingly recognized as a vital component of health care education owing to its ability to bridge disciplinary divides and cultivate in participants the essential collaborative competencies necessary to address complex patient care challenges [1,2]. By fostering teamwork, communication, and mutual role understanding among individuals in diverse health care disciplines, IPE addresses gaps traditionally reinforced by siloed curricula [1,3]. Empirical evidence has consistently demonstrated that early exposure to IPE enhances undergraduate health care students’ teamwork abilities, communication skills, and readiness for collaborative practice [4,5]. Conversely, the absence of IPE can limit students’ interprofessional skills, potentially leading to poorer health outcomes for patients in the future [6]. However, despite its proven benefits, IPE implementation faces persistent systemic barriers [7,8].

Geographical and institutional barriers often limit meaningful interaction among individuals in different health care disciplines, as many educational programs are housed in separate departments or institutions [9]. This separation fosters professional silos, hindering students’ ability to develop effective cross-disciplinary communication and collaboration skills. Furthermore, faculty’s resistance to change, particularly among those accustomed to traditional, discipline-centered teaching methods, presents an additional challenge [10,11]. Compounding these issues is the frequent absence of established frameworks or guidelines for IPE integration into existing curricula. Without systematic implementation, IPE initiatives risk being reduced to superficial additions rather than becoming foundational components of health care education [6,12]

To enhance learning outcomes in IPE, several strategies have been implemented. A systematic review revealed that the most commonly used teaching and learning methods in universities to implement IPE were problem-based learning, case-based learning, and team-based learning [13]. In addition, simulation-based learning, e-learning, and serious games have been recognized as effective methods for cultivating IPE competencies [13-16]. Technology enables flexible learning options offered by IPE and can accommodate many schedules and geographical distances. This accessibility allows students from many fields to engage in group learning projects independent of physical limitations [17].

### Technology-Enabled IPE: Serious Games as a Solution

Serious games are defined as interactive digital tools designed to merge gameplay with measurable learning outcomes. They have emerged as a promising solution to IPE implementation challenges [18]. These games can be delivered through 2 distinct modalities, namely immersive virtual reality (IVR) and web-based platforms.

Incorporating IVR into IPE can provide students with repeatable experiential learning opportunities within and across disciplines. It provides high-fidelity clinical simulations that enhance experiential learning through realistic scenarios, emotional presence, and embodied cognition [19]. IVR’s unique capacity for perspective-taking and risk-free practice makes it particularly valuable for empathy development and high-stakes skills training [20]. IVR can overcome organizational barriers, such as a lack of time, limited skilled professional instructors, and a lack of space [21,22], as all of the scenarios are predesigned and set in an IVR environment.

Web-based platforms emphasize accessibility, flexibility, and scalability, enabling asynchronous collaboration and real-time performance tracking for geographically dispersed learners. Their strength lies in creating interactive digital spaces that support interprofessional interaction without temporal or spatial constraints [23]. This allows students to engage in activities at their convenience from home, offering multiple opportunities for practice. Additionally, it overcomes geographical and institutional challenges, such as limited space and resources [23].

Both modalities share the core benefits of serious games, which improve engagement, knowledge retention, and collaborative skills [24]. IVR-based serious games offer unique advantages in creating authentic health care team dynamics through immersive simulations [25]. Studies indicate that these environments significantly enhance collaborative problem-solving and interprofessional learning experiences [26]. The technology’s unparalleled capacity for experiential immersion accelerates the development of competency in complex interpersonal skills by fostering teamwork and enhancing knowledge acquisition [19].

Meanwhile, studies have consistently demonstrated that interdisciplinary collaboration facilitated by web-based serious games enables students to consolidate and apply knowledge more effectively, fostering collaborative behaviors that enhance clinical decision-making skills [27,28]. The inherent accessibility and lower infrastructure requirements of these arrangements make them particularly suitable for resource-constrained settings [23].

Despite these established benefits, no studies have directly compared how these delivery modalities (IVR vs web-based learning) influence IPE outcomes. This gap leaves educators without the evidence necessary to choose between high-immersion (but resource-intensive) IVR and scalable (but less immersive) web-based platforms.

In addition, the successful implementation of technology-mediated IPE hinges on 2 critical factors: sustained student engagement and meaningful interpersonal connections [29]. These elements are particularly vulnerable in digital learning environments, where the lack of physical proximity may compromise the development of interdisciplinary relationships. This challenge is especially pronounced in web-based platforms, which must overcome the inherent limitations of asynchronous interaction to foster authentic collaboration [30]. Conversely, IVR offers unique advantages in this regard, with its capacity to generate emotional presence and embodied learning experiences that may strengthen interprofessional bonds [20].

This study addresses these considerations through a comprehensive comparison of 2 technological approaches. We explore how experiential learning, utilizing IVR and web-based platforms, differentially influences both concrete learning outcomes and essential experiential factors. Specifically, the investigation evaluates their relative effectiveness in developing interprofessional competencies (teamwork and knowledge acquisition) while simultaneously cultivating the motivation (interest and competence) that predicts ongoing engagement with IPE collaboration [31] and the community-building aspects (membership and emotional ties) that ensure long-term engagement with collaborative practice [32].

### Objectives

This study compared IVR with web-based serious games for developing health care students’ (1) interprofessional competencies (teamwork readiness and knowledge acquisition) and (2) learning experiences (motivation and community perception), with the aim of identifying the optimal modality for IPE implementation. The investigation specifically assessed how each approach differentially impacts competency development, intrinsic engagement (interest and perceived competence), and community formation (membership, support, and connection), providing evidence for technology-enhanced IPE strategies.

## Methods

### Patient and Public Involvement

Since this study aimed to compare the effectiveness of IVR and game-based learning in IPE among health care students and was conducted in a local university, there was no patient or public involvement.

### Trial Design

This study used a multimethod design, which incorporated pre/postlearning approaches to measure competency gains, knowledge-checking games to evaluate the application of skills, and focus group interviews to capture qualitative experiential differences between the 2 delivery modalities. This comprehensive approach enabled a robust comparative analysis of the educational effectiveness of the 2 modalities, addressing a critical gap in understanding how technological immersion levels impact IPE outcomes [33]. This study has been reported in accordance with the CONSORT (Consolidated Standards of Reporting Trials) 2025 checklist (Checklist 1) and CONSORT-EHEALTH checklist for randomized controlled trials (Checklist 2) [34,35]. In line with these guidelines, the study was retrospectively registered at ClinicalTrials.gov on February 27, 2026 (NCT07474701). This was an educational randomized study conducted among undergraduate health care students, with outcomes focused on training-related measures that included knowledge, attitudes, skills, and learning outcomes rather than patient health outcomes. Given the low-risk, nonclinical nature of the study and outcomes, the trial was not prospectively registered.

### Trial Setting and Eligibility Criteria

This study was conducted at a government-funded university that offers undergraduate nursing and physiotherapy programs. Convenience sampling was used to recruit undergraduate health care students from the 2024/25 cohort. The participant pool consisted of 215 third-year nursing students enrolled in a gerontological nursing course and 68 third- and fourth-year physiotherapy students attending preclinical training workshops.

All eligible students were invited to complete surveys about their learning experiences as part of the course evaluation. However, only students who provided consent were included in the study analysis and reporting. Students were organized into 34 interprofessional groups, each comprising 7‐8 nursing students and 1‐2 physiotherapy students, and they were randomly allocated to IVR training (17 IPE groups) or web-based serious gaming (17 IPE groups). Details of the randomization procedure are provided in the Randomization section.

Participation in focus groups was optional. After completion of the IPE learning activities, 4 face-to-face focus group discussions were conducted, with each session lasting an average of approximately 60 minutes. Representatives from the 34 groups were invited to participate in the focus group (34 students; 22 nursing students and 12 physiotherapy students).

### Ethical Considerations

Ethical approval was obtained from the Human Subjects Ethics Review Committee of the university (HSEARS20240827002). While participation in the IPE tutorials was mandatory for nursing and physiotherapy students, involvement in the research study was strictly voluntary. Students were explicitly assured that their decision to participate would not affect their academic standing, course grades, or access to learning opportunities. No monetary or nonmonetary compensation, course credits, or other incentives were provided for participation in the research study. Written informed consent was obtained from all study participants, and stringent confidentiality measures were implemented, including deidentification of all data prior to analysis. Access to raw data was restricted to only 3 nursing faculty members (JYWL, PPKK, and SW), the physiotherapy lecturer (CKHW), and the project research assistant (RWYW) to protect participant privacy while enabling necessary educational assessments. This clear separation between required learning components and optional research participation was maintained throughout the study period, with all students receiving equal access to learning experiences regardless of their research participation status. In addition, no individual participants in this study can be identified from the images included in the manuscript or supplementary materials.

### Intervention and Comparator: IPE Program Design

**Figure 1.**
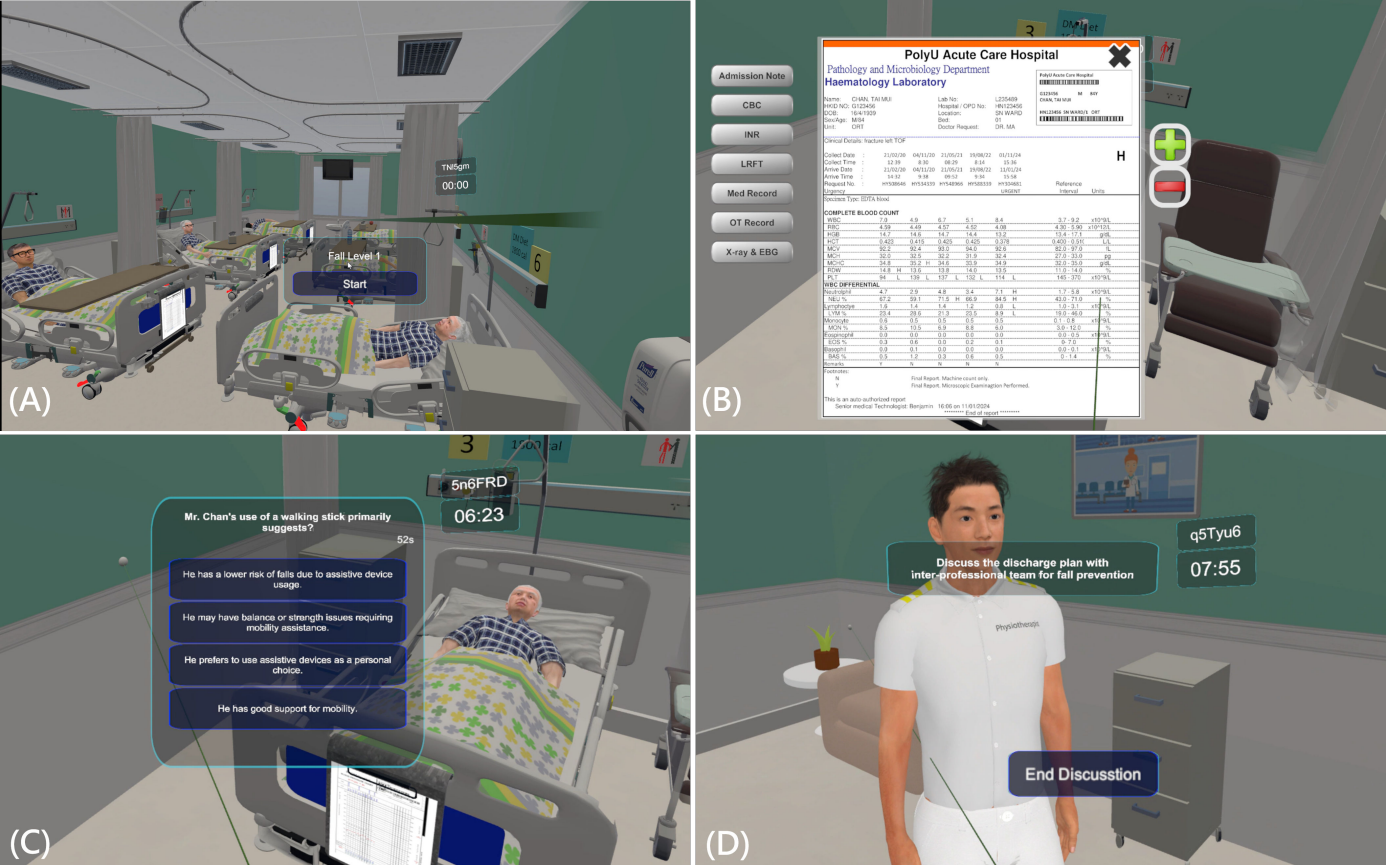
Interface screenshots of the immersive virtual reality platform, including (A) the overall virtual environment settings, (B) patient information with case details, (C) knowledge-checking questions, and (D) a discussion panel from the first-person view of a nurse.

**Figure 2.**
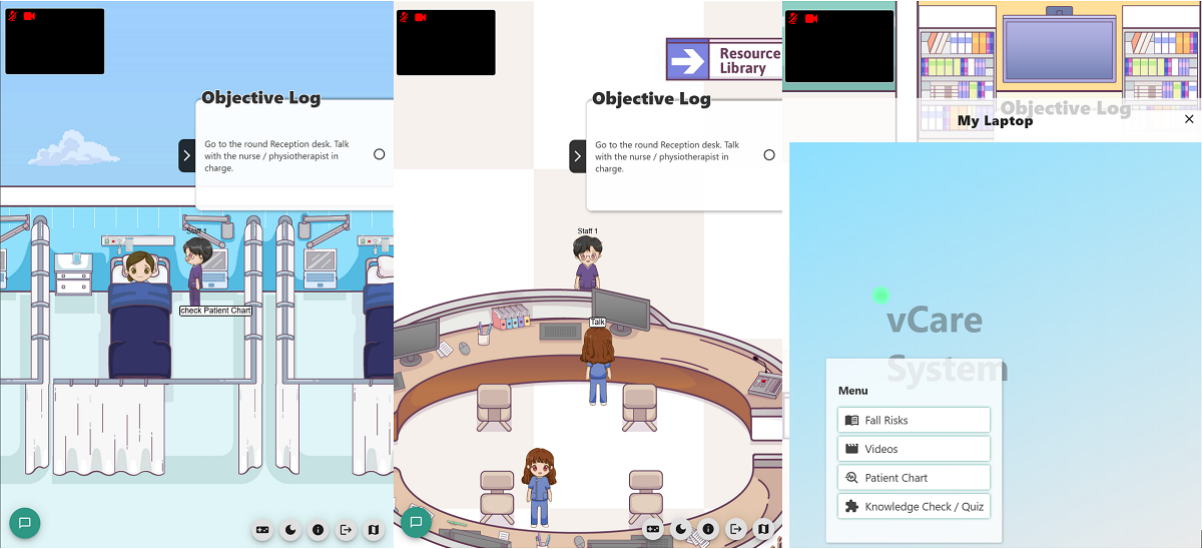
Interface screenshots of the web-based platform, including the overall environment settings (left and middle) and the resource hub with knowledge-checking questions and self-learning materials (right).

The IPE learning activities were strategically implemented during weeks 7‐8 of the 13-week semester to ensure that the students would have acquired foundational knowledge before engaging in collaborative competency development. The research team designed a unified serious game flow that simulated the care trajectory of an older adult recovering from a fall-induced hip fracture, with the following three IPE-focused levels: (1) interprofessional care transition (acute to rehabilitation), (2) interprofessional team dynamics (rehabilitation ward), (3) interprofessional discharge coordination (community reintegration). This identical clinical scenario was delivered through both modalities—IVR (Figure 1) and web-based platform (Figure 2)—maintaining consistency in learning objectives across conditions. The web-based platform had different user interface panels for the PC and mobile displays, with a website URL that made both the PC and mobile platforms user-friendly. The 2 formats differed only in their technological delivery and gamification elements, with content that did not require hands-on practice (Table 1).

**Table 1.** Overview of the learning objectives and activities of interprofessional education–focused levels and comparisons of gamification elements between IVR^a^ and web-based platforms.

Variable	Interprofessional care transition (acute to rehabilitation)	Interprofessional team dynamics (rehabilitation ward)	Interprofessional discharge coordination (community reintegration)
Learning objective	Develop shared decision-making skills for transitional care planning.	Practice role clarity and communication in fracture rehabilitation.	Refine patient-centered discharge strategies.
Learning activities	Nursing and physiotherapy students collaborate on (1) transferring documentation and medication reconciliation, (2) setting shared rehabilitation goals, and (3) addressing potential complications (eg, delirium risk [nursing focus] and early mobilization [physiotherapy focus]).	Students coordinate daily routines to resolve real-world interdisciplinary challenges (eg, balancing wound care schedules [nursing] with mobility exercises [physiotherapy]) and practice communication and teamwork to ensure patient-centered care.	Interprofessional teams work together to (1) develop comprehensive discharge plans, (2) address medication management (nursing) and home mobility adaptations (physiotherapy), and (3) apply structured negotiation for patient transition to home care.
Gamification elements of the IVR platform	—^b^	Real-time verbal discussions within a virtual ward, with all audio interactions automatically recorded for teaching and review purposes.	—
Gamification elements of the web-based platform	—	Separated real-time discussion rooms using text or video chat on the platform for group discussions, with all sessions recorded in the cloud and archived for teaching and review purposes.	—

a IVR: immersive virtual reality.

b Not applicable.

### Implementation

The IPE program was delivered through 2 modalities. Quick informal briefing and debriefing sessions lasting 30 minutes were conducted with the participants. Seventeen IVR groups participated in a structured 2-hour face-to-face tutorial during the 7th week of the 13-week semester. The tutorial used 5 IVR setups to simulate clinical rehabilitation environments, where 4-5 IPE teams per tutorial rotated through game-based learning activities in a similar setup during each session. Meanwhile, the web-based groups accessed identical content through a web-based platform over the same week, at their convenience, enabling asynchronous collaboration without time or location constraints.

### Harms

The potential harms associated with the intervention were minimal. One possible adverse effect was transient dizziness or nausea (ie, virtual reality sickness) associated with prolonged IVR use. Both students and lecturers were informed of this risk. Students were instructed to stop the activity immediately and report any physiological discomfort to the lecturers so that appropriate support could be provided. If symptoms occurred, students were allowed to take a break or withdraw from the session as needed.

### Sample Size

A power analysis determined the sample size. Previous studies of similar educational interventions reported medium to large effect sizes in learning outcomes from pre- to postintervention [36-38]. Adopting a conservative approach, we based our power analysis on detecting a medium effect using a 2×2 mixed multivariate analysis of variance (MANOVA). The results indicated that a sample size of 128 would be sufficient to detect a medium 2-tailed effect at a significance level of *P*<.05 with 80% power. We anticipated a sample size of around 150‐160 to account for potential missing data. Based on the nurse-physiotherapist ratio, this would translate to approximately 112‐128 student nurses and 32‐38 student physiotherapists.

### Outcomes

#### Overview

Participants were assessed at 2 strategic timepoints to capture both modality-specific and comparative learning outcomes. Following completion of their first assigned learning approach (either IVR or the web-based platform) but prior to experiencing the alternative modality, all participants completed self-administered surveys where the knowledge-checking activities were embedded in the IPE learning games. This timing allowed for the independent evaluation of the discrete learning experiences and outcomes associated with each platform. Subsequently, after all groups had engaged with both teaching approaches through the crossover design, we conducted 4 focus group discussions. This later timing enabled the participants to provide informed comparative reflections on the relative strengths and limitations of both modalities, having gained first-hand experience with each approach. The phased assessment strategy thus yielded both platform-specific and holistic evaluation data for a comprehensive understanding of the educational innovations.

#### Demographic Data

Demographic variables included age, gender, profession (nursing/physiotherapy), course, experience with online games, and total play time per day.

#### Primary Outcomes

The Readiness for Interprofessional Learning Scale (RIPLS) was used to evaluate the students’ preparedness to engage in collaborative IPE. This scale consists of 19 items organized into three factors that assess students’ views on: (1) teamwork and collaboration, (2) professional identity, and (3) professional roles and responsibilities [39]. Responses are measured using a 5-point Likert scale (1=strongly disagree, 2=somewhat disagree, 3=neither agree nor disagree, 4=somewhat agree, and 5=strongly agree). Higher scores on the total scale and its subscales indicate increased readiness and more favorable attitudes on the importance of IPE and collaborative practice among health care students [40]. The overall RIPLS demonstrated excellent internal consistency, with a reliability coefficient (Cronbach α) of 0.90 [39,40].

The Brief Sense of Community Scale (BSCS) was used to evaluate the participants’ psychological sense of community while playing the games [39,41]. It consists of 18 items measuring 4 dimensions: needs fulfillment (the belief that community members will help each other), membership (a sense of belonging to the community), influence (a sense of contributing to the community), and emotional connection (a shared emotional connection/experience) [41]. A 5-point Likert-type scale, with 1 denoting “strongly disagree” and 5 denoting “strongly agree,” was used by participants to answer the BSCS items. A stronger sense of community is indicated by a higher BSCS score [39]. The scale has demonstrated excellent internal consistency (Cronbach *α*=0.92) [39].

The Intrinsic Motivation Inventory (IMI) questionnaire was used to evaluate different aspects of the students’ motivation in relation to the learning approaches [42]. Nine items distributed over 3 subscales were used: interest/enjoyment, perceived competence, and pressure/tension. Interest/enjoyment represents a self-reported measure of intrinsic motivation (the level of interest and joy felt while performing a certain activity). Perceived competence is considered a positive predictor of intrinsic motivation. Perceived competence involves assessing participants’ feelings when they are carrying out a task. The pressure/tension subscale, which evaluates whether participants experience pressure to do well during an activity, is thought to be a negative predictor [42]. This version consists of 9 items scored on a Likert scale ranging from 1 (not at all true) to 7 (very true). The Cronbach α coefficients for the interest/enjoyment, perceived competence, and pressure/tension subscales were 0.78, 0.80, and 0.68, respectively, demonstrating generally adequate internal consistency [42]. The overall IMI scale has shown strong internal consistency, with a Cronbach α value of 0.85 [42].

Multiple-choice questions (MCQs) were used to measure students’ learning outcomes. The questions and answers were collaboratively developed by academics from the departments of nursing and rehabilitation sciences. A total of 20 questions were developed (10 related to discipline-specific knowledge and 10 related to knowledge of IPE). See Multimedia Appendix 1 for the MCQs that were developed and used to measure students’ learning outcomes.

### Randomization

Students were organized into 34 interprofessional groups, each comprising 7‐8 nursing students and 1‐2 physiotherapy students. Randomization was conducted at the group level for administrative purposes. A computer-generated random sequence (1:1 allocation) was used to allocate the 34 IPE groups to either IVR training (17 IPE groups) or web-based serious gaming (17 IPE groups). The allocation sequence was generated and implemented by the lecturers (JYWL and CKHW). Owing to the nature of the study design, students were aware of the groups to which they had been assigned. Following a crossover design, the groups used their initial learning modality for 1 week before switching to the alternative modality. All students were invited to complete surveys about their learning experiences, and participation in the focus groups was optional. As the surveys and knowledge-checking tests (MCQs) were self-administered, blinding was not required.

### Statistical Methods

#### Quantitative Data

IBM SPSS 29.0 software was used to analyze the quantitative data. With regard to descriptive statistics, mean and SD were used for continuous data, and frequency and percentage were used for categorical variables. A chi-square test or Fisher exact test was used for categorical variables. According to the trial protocol, a mixed 2×2 MANOVA was prespecified to explore the interactions between group differences and the within-subject factor. However, upon examining the data, the assumption of normality was violated. Consequently, as a deviation from the prespecified analysis, nonparametric tests were used. The median and IQR were used to present outcome measures. Within-group differences were analyzed using the Wilcoxon signed rank test, while between-group differences were analyzed using the Mann-Whitney *U* test. To strengthen the interpretation of the results, effect sizes (*r*) and CIs were used. The level of significance was set at .05. Samples with missing data were excluded from the analysis.

#### Qualitative Analysis

Qualitative data were collected through focus group interviews. The semistructured interviews, which used overarching questions, were transcribed verbatim and analyzed using thematic analysis (see Multimedia Appendix 2 for the interview questions). The process of analysis began with initial open coding, during which significant statements and phrases were identified for further thematic development. These codes were subsequently grouped with meaningful subthemes. Lastly, the subthemes were condensed to form overarching themes, leading to the identification of key themes. To ensure qualitative rigor, all interview transcripts were independently coded by 2 researchers (RWYW and JYWL). The initial codes were compared and discussed to ensure consistency, and any discrepancies that could not be resolved were reviewed by a third researcher (KC) to reach a consensus. The development of key themes followed a similar iterative process involving comparison, discussion, and consensus among the 2 researchers (RWYW and JYWL) to ensure the credibility and trustworthiness of the findings.

## Results

### Participant Flow and Recruitment

**Figure 3.**
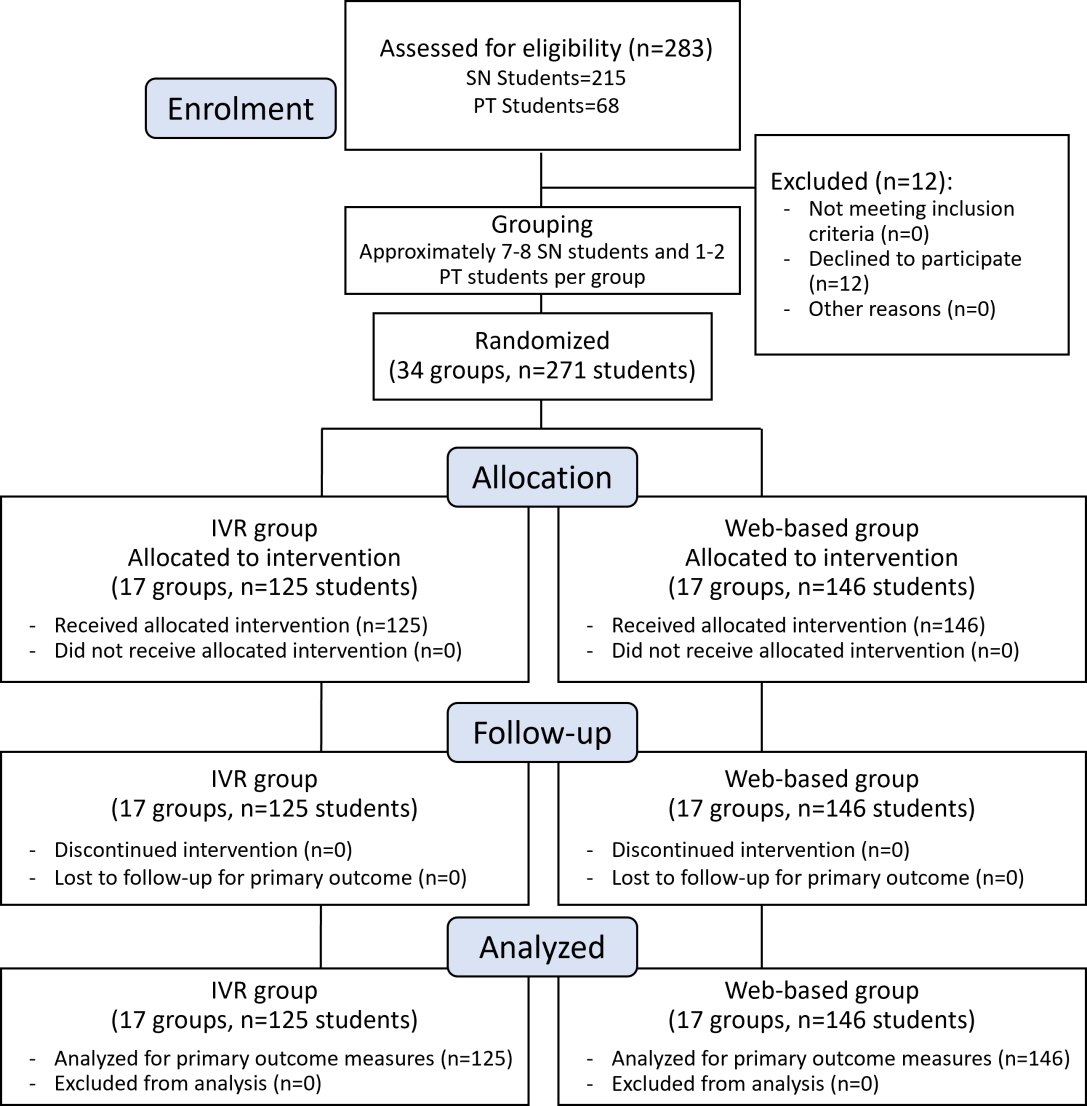
Participant flowchart. IVR: immersive virtual reality; PT: physiotherapy; SN: School of Nursing.

A total of 283 students were invited to participate. Of these, 271 students (125 in the IVR group and 146 in the web-based group) consented to join the study, yielding a response rate of 95.8% (271/283). Participant flow through the trial is shown in Figure 3. The final sample consisted of 203 nursing students and 68 physiotherapy students.

### Baseline Data

The majority of participants were female (160/271, 59.0%), were aged between 19 and 21 years (173/271, 63.8%), and had experience with online gaming (250/271, 92.3%). Significant differences in age distribution (*P*=.001) and course enrollment (*P*<.001) were noted between the IVR and web-based groups (Table 2). There were no significant differences in gender, profession, online gaming experience, or daily playtime (*P*≥.05). In addition, no significant between-group differences were identified for the outcome measures at baseline (*P*≥.05; Multimedia Appendix 3).

**Table 2.** Demographic data of the participants in the IVR^a^ and web-based groups.

Characteristic	IVR group (n*=*125), n (%)	Web-based group (n*=*146), n (%)	Chi-square (*df*)	*P* value
Gender			2.4 (1)	.12
Male	45 (36.0)	66 (45.2)		
Female	80 (64.0)	80 (54.8)		
Age (years)			13.4 (2)	.001
≤18	0 (0.0)	0 (0.0)		
19‐21	79 (63.2)	94 (64.4)		
22‐24	27 (21.6)	47 (32.2)		
≥25	19 (15.2)	5 (3.4)		
Profession			2.5 (1)	.11
Nursing	88 (70.4)	115 (78.8)		
Physiotherapy	37 (29.6)	31 (21.2)		
Course			38.6 (3)	<.001
SN^b^ 53445-SY	4 (3.2)	23 (15.8)		
SN 53445	61 (48.8)	91 (62.3)		
SN 53081	23 (18.4)	1 (0.7)		
RS^c^ 37500	37 (29.6)	31 (21.2)		
Experience with online games			0.4 (1)	.55
Yes	114 (91.2)	136 (93.2)		
No	11 (8.8)	10 (6.8)		
Total play time per day (hours)			4.4 (2)	.11
<1	73 (58.4)	70 (47.9)		
1‐10	51 (40.8)	71 (48.6)		
>10	1 (0.8)	5 (3.4)		

a IVR: immersive virtual reality.

b SN: School of Nursing.

c RS: Department of Rehabilitation Sciences.

### Analyzed Outcomes and Estimation for Quantitative Outcomes

#### Primary Outcomes

##### Within-Group Comparisons of RIPLS, BSCS, IMI, and MCQ Scores in the IVR Group

No significant differences were noted in RIPLS scores (*P*=.24). Participants demonstrated significant improvements in BSCS scores (*P*=.001), scores of the “pressure/tension” subscale of the IMI (*P*<.001), and scores of the knowledge-checking MCQs (*P*<.001; Table 3). Significant increases were noted in total BSCS scores (*r*=−0.29, 95% CI 0.06-0.31; *P*=.001) and the scores of the dimensions of “influence” (*r*=−0.32, 95% CI 0.00-0.50; *P*<.001) and “emotional connection” (*r*=−0.33, 95% CI 0.25-0.50; *P*<.001; Multimedia Appendix 4). Significant decreases were noted in the scores of the “pressure/tension” subscale of the IMI (*r*=−0.45, 95% CI −0.05 to –0.25; *P*<.001). Moreover, there were significant improvements in the scores of the knowledge-checking MCQs (*r*=−0.64, 95% CI 2.00-3.00; *P*<.001; Table 3).

**Table 3.** Within-group pre-post comparisons of primary outcome measures in the IVR^a^ group (n=125).

Outcome measure		Pre, median (IQR)	Post, median (IQR)	95% CI^b^	Effect size^c^ (*r*)	*z* score	*P* value^d^
RIPLS^e^^,^^f^							
Total score		3.58 (3.42‐3.84)	3.58 (3.37‐3.79)	−0.11 to 0.03	−0.10	−1.17	.24
BSCS^g^^,^^h^							
Total score		3.38 (3.00‐3.88)	3.63 (3.19‐4.00)	0.06 to 0.31	−0.29	−3.21	.001^i^
IMI^j^^,^^k^							
Interest/enjoyment		4.00 (4.00‐4.60)	4.20 (3.80‐5.00)	−0.10 to 0.20	−0.05	−0.57	.57
Perceived competence		4.00 (4.00‐4.50)	4.00 (3.50‐5.00)	0.00 to 0.25	−0.17	−1.93	.05
Pressure/tension		4.00 (3.50‐4.00)	3.50 (2.50‐4.00)	−0.05 to −0.25	−0.45	−5.07	<.001^i^
MCQs^l^^,^^m^							
Total score		13.00 (9.00‐15.00)	15.00 (13.00‐17.00)	2.00 to 3.00	−0.64	−7.15	<.001^i^

a IVR: immersive virtual reality.

b Hodges-Lehmann CI for the median paired difference (posttest minus pretest scores).

c Effect size=z/√n.

d Wilcoxon signed rank test.

e RIPLS: Readiness for Interprofessional Learning Scale.

f An increase in the score indicates a more positive attitude.

g BSCS: Brief Sense of Community Scale.

h An increase in the score indicates a stronger sense of community.

i Statistically significant difference.

j IMI: Intrinsic Motivation Inventory.

k An increase in the score indicates an increase in motivation and experience.

l MCQs: multiple-choice questions.

m An increase in the score indicates clearer knowledge.

##### Within-Group Comparisons of RIPLS, BSCS, IMI, and MCQ Scores in the Web-Based Group

No significant differences were noted in RIPLS scores (*P*=.90). Participants demonstrated significant improvements in BSCS scores (*P*=.001), scores of all 3 subscales of the IMI (*P*<.05), and scores of the knowledge-checking MCQs (*P*<.001; Table 4). Significant increases were seen in total BSCS scores (*r*=−0.27, 95% CI 0.07-0.31; *P*=.001) and the scores of the dimensions of “influence” (*r*=−0.30, 95% CI 0.00-0.50; *P*<.001), “emotional connection” (*r*=−0.24, 95% CI 0.00-0.50; *P*=.004), and “needs fulfillment” (*r*=−0.18, 95% CI 0.00-0.50; *P*=.03; Multimedia Appendix 4). Regarding the IMI, significant increases were noted in the scores of the “interest/enjoyment” subscale (*r*=−0.17, 95% CI 0.00-0.30; *P*=.043) and “perceived competence” subscale (*r*=−0.19, 95% CI 0.00-0.50; *P*=.02), while significant decreases were noted in the scores of the “pressure/tension” subscale (*r*=−0.46, 95% CI −0.75 to −0.50; *P*<.001). Moreover, there were significant improvements in the scores of the knowledge-checking MCQs (*r*=−0.80, 95% CI 4.50-6.00; *P*<.001; Table 4).

**Table 4.** Within-group pre-post comparisons of primary outcome measures in the web-based group (n=146).

Outcome measure		Pre, median (IQR)	Post, median (IQR)	95% CI^a^	Effect size^b^ (*r*)	*z* score	*P* value^c^
RIPLS^d^^,^^e^							
Total score		3.63 (3.47‐3.86)	3.63 (3.42‐3.89)	−0.05 to 0.05	−0.01	−0.13	.90
BSCS^f^^,^^g^							
Total score		3.44 (3.00‐4.00)	3.75 (3.38‐4.00)	0.07 to 0.31	−0.27	−3.27	.001^h^
IMI^i^^,^^j^							
Interest/enjoyment		4.20 (4.00‐4.56)	4.60 (4.00‐5.25)	0.00 to 0.30	−0.17	−2.03	.043^h^
Perceived competence		4.00 (4.00‐4.50)	4.00 (4.00‐5.00)	0.00 to 0.50	−0.19	−2.29	.02^h^
Pressure/tension		4.00 (3.88‐4.00)	3.50 (3.00‐4.00)	−0.75 to −0.50	−0.46	−5.58	<.001^h^
MCQs^k^^,^^l^							
Total score		12.00 (8.00‐14.00)	18.00 (15.00‐19.00)	4.50 to 6.00	−0.80	−9.63	<.001^h^

a Hodges-Lehmann CI for the median paired difference (posttest minus pretest scores).

b Effect size=z/√n.

c Wilcoxon signed rank test.

d RIPLS: Readiness for Interprofessional Learning Scale.

e An increase in the score indicates a more positive attitude.

f BSCS: Brief Sense of Community Scale.

g An increase in the score indicates a stronger sense of community.

h Statistically significant difference.

i IMI: Intrinsic Motivation Inventory.

j An increase in the score indicates an increase in motivation and experience.

k MCQs: multiple-choice questions.

l An increase in the score indicates clearer knowledge.

##### Comparisons of RIPLS, BSCS, IMI, and MCQ Scores Between the IVR and Web-Based Groups

The web-based group demonstrated superior performance in knowledge-checking MCQs compared with the IVR group (IVR group median score: 15.00, web-based group median score: 18.00; *r*=−0.34, 95% CI −3.00 to −2.00; *P*<.001). However, no significant differences were observed between the IVR and web-based groups in the total scores of the RIPLS, BSCS, or IMI at the postassessment stage (Table 5).

**Table 5.** Between-group comparisons of primary outcome measures after the intervention.

Outcome measure	IVR^a^ group, median (IQR)	Web-based group, median (IQR)	95% CI^b^	Effect size^c^ (*r*)	*z* score	*P* value^d^
RIPLS^e^^,^^f^						
Total score	3.58 (3.37‐3.79)	3.63 (3.42‐3.89)	−0.16 to 0.00	−0.10	−1.60	.11
BSCS^g^^,^^h^						
Total score	3.63 (3.19‐4.00)	3.75 (3.38‐4.00)	−0.25 to 0.00	−0.09	−1.48	.14
IMI^i^^,^^j^						
Interest/enjoyment	4.20 (3.80‐5.00)	4.60 (4.00‐5.25)	−0.40 to 0.00	−0.09	−1.43	.15
Perceived competence	4.00 (3.50‐5.00)	4.00 (4.00‐5.00)	−0.50 to 0.00	−0.06	−1.03	.30
Pressure/tension	3.50 (2.50‐4.00)	3.50 (3.00‐4.00)	0.00 to 0.00	−0.02	−0.32	.75
MCQs^k^^,^^l^						
Total score	15.00 (13.00‐17.00)	18.00 (15.00‐19.00)	−3.00 to −2.00	−0.34	−5.53	<.001^m^

a IVR: immersive virtual reality.

b Hodges-Lehmann CI for the median paired difference.

c Effect size=z/√n.

d Mann-Whitney *U* test.

e RIPLS: Readiness for Interprofessional Learning Scale.

f An increase in the score indicates a more positive attitude.

g BSCS: Brief Sense of Community Scale.

h An increase in the score indicates a stronger sense of community.

i IMI: Intrinsic Motivation Inventory.

j An increase in the score indicates an increase in motivation and experience.

k MCQs: multiple-choice questions.

l An increase in the score indicates clearer knowledge.

m Statistically significant difference.

### Ancillary Analyses for Qualitative Results

#### Focus Group Participants

A total of 34 participants were engaged in the focus groups, and most of them were female (24/34, 71%), were aged 19‐21 years (23/34, 68%), were studying nursing (24/34, 71%), had experience with online games (23/34, 68%), and had no prior experience with IPE (28/34, 82%; Table 6). The average rating for overall experience was 5.78 (SD 1.97) out of 10 points, and the ratings for IVR and web-based learning were 6.03 (SD 2.24) and 5.47 (SD 2.13), respectively.

**Table 6.** Demographic data of the participants in the focus groups.

Characteristic	Value (n*=*34)
Gender, n (%)	
Male	10 (29)
Female	24 (71)
Age (years), n (%)	
≤18	0 (0)
19‐21	23 (68)
22‐24	9 (27)
≥25	2 (6)
Profession, n (%)	
Nursing	24 (71)
Physiotherapy	10 (29)
Course, n (%)	
SN^a^ 53445-SY	5 (15)
SN 53445	19 (56)
SN 53081	0 (0)
RS^b^ 37500	10 (29)
Experience with online games, n (%)	
Yes	23 (68)
No	11 (32)
Year of study, n (%)	
Year 1	0 (0)
Year 2	3 (9)
Year 3	7 (21)
Year 4	23 (68)
Year 5	1 (3)
Prior experience with interprofessional education, n (%)	
Yes	6 (18)
No	28 (82)
Rating of experience, mean (SD)	
Overall experience	5.78 (1.97)
IVR^c^	6.03 (2.24)
Web-based	5.47 (2.13)

a SN: School of Nursing.

b Department of Rehabilitation Sciences.

c IVR: immersive virtual reality.

#### Qualitative Outcomes: Focus Groups

Four focus group interviews were conducted, and they included a total of 34 participants engaged with both the IVR and web-based platforms. Six themes and 17 subthemes were identified, and they, along with quotations, are summarized in Table 7. The themes included (1) recognizing value, gaps, and opportunities for deeper collaboration; (2) shared learning, trust, and emotional connection across disciplines; (3) engagement, autonomy, and authenticity in digital learning environments; (4) enhancing knowledge retention; (5) balancing innovation with implementation; and (6) enhancing realism and inclusivity. Data saturation was achieved, as all 4 focus groups yielded consistent themes with no new substantive insights emerging across groups, indicating that the qualitative data adequately captured the range of student experiences across both learning modalities.

**Table 7.** Thematic analysis of the focus group semistructured interview.

Themes and subthemes	Quotes^a^
1. Recognizing value, gaps, and opportunities for deeper collaboration	
1a. Recognition of the value of interprofessional collaboration	We rarely have interdisciplinary practice, so this is a great opportunity to understand each other’s responsibilities, perspectives, and thoughts. [Student #13]
1b. Desire for deeper role integration	In IVR^b^, both PT^c^ and SN^d^ students can play separately without many tasks needing to be completed together. Adding some content where those from two professions need to think and analyze together would help in understanding each other’s roles and how they complement each other. [Student #37]
1c. Perceived lack of collaboration in practice	The collaboration wasn’t very extensive. For example, during bed notes, we each handled our own parts, so I don’t think it was very helpful for collaboration. [Student #42]
1d. Positive learning through interaction	Because previously, there were very few opportunities to engage in discussions like this with people from other professions. Usually, it was just with my own classmates. This time, it was the first time to collaborate on a project with nursing students, so I learned during the process. Apart from communication, we exchanged knowledge about our respective professions. [Student #15]
2. Shared learning, trust, and emotional connection across disciplines	
2a. A shared learning structure enhances connection for web-based learning	I think I might prefer the web-based platform because I feel like I can learn more from it. There are more multiple-choice questions, mini games, and we go through the whole flow together. There are maybe six or seven checkpoints, which helps relate the content back to what we learned in class. [Student #211]
2b. Interdisciplinary learning enriches understanding	Because we are working with people from different disciplines, you get to see more perspectives, for example to see how PT students experience things differently. Sometimes they even teach us how to analyze a problem or a case where nursing students might skip parts not directly related to our duty. But with PT students involved, you end up learning more. [Student #28]
2c. Collaborative problem-solving builds trust	It felt like teamwork when everyone was looking at the same case together. That created a nice sense of bonding, and we could immediately catch things that others might have missed. [Student #11]
2d. Supportive communication fosters engagement	What I liked most was being able to discuss things as a group. If you didn’t understand something, you could just ask others, and they would help you. Through discussion, you gained a deeper understanding of each case, which made me feel more engaged. [Student #11]
3. Engagement, autonomy, and authenticity in digital learning environments	
3a. Engagement through playful design	There are many types and numbers of mini-games, making a web-based game less difficult or tiring to play, which is more interesting. [Student #44]
3b. Empowered and self-directed learning	I think the web-based game allows us to explore more on our own because it is an online platform. We have more preparation time, can learn new knowledge instantly, and get immediate feedback. If we don’t understand something, we can ask in lectures later, and there are checkpoints for checking our progress, which is better for learning. [Student #212]
3c. Authenticity enhances value	I think that the setting, elements, and process are very similar to a hospital setting. [Student #13]
4. Enhancing knowledge retention	
4a. Challenge enhances learning depth for IVR	Those students who were playing the IVR had a time limit that forced them to think quickly. So, it might make the memory deeper, and we might actually learn more things. [Student #14]
4b. Practice and feedback support web-based learning	Because the web-based platform allows you to play the games many times, it is like practicing exam papers repeatedly. If you play a few more times, you will remember. But in IVR, if you make a mistake, you will only be told that the answer is wrong and then need to move on to the next question. However, with web-based learning, the memory points are deeper. [Student #43]
5. Balancing innovation with implementation	
5a. Appreciation for a creative and engaging instructional design	I understand that the SN and PT teachers worked hard to provide a new experience, making it more interesting than just attending tutorials. [Student #37]
5b. Technical barriers disrupt learning continuity	The connection was very poor, groupmates kept disconnecting, and it wasn’t like a normal conference. [Student #31]
6. Enhancing realism and inclusivity	
6a. Expanding interprofessional scope for holistic learning	I think people from more professions should be included, such as doctors and social workers, to make a more comprehensive care plan. [Student #28]
6b. Enhancing the simulation of realism through patient interactions	I think patient reactions should be added. Currently, we can only understand the patient’s condition through the layout, but in reality, we would at least ask the patient. [Student #42]

a Quotes from focus group participants are attributed using anonymized identification codes based on transcription order.

b IVR: immersive virtual reality.

c PT: physiotherapy.

d SN: School of Nursing.

The focus group interviews were performed to evaluate the students’ views on the IPE program in relation to the outcome measures (Table 7). With regard to readiness for interpersonal learning, the students expressed appreciation for the opportunities to engage in interprofessional learning, particularly when such experiences had previously been limited. They recognized the value of understanding different professional roles but also highlighted the need for more integrated tasks that require genuine collaboration. Positive reflections on learning through interaction suggest that when collaboration is meaningful, it enhances both engagement and professional understanding.

The sense of community was strengthened through shared learning structures, interdisciplinary dialogue, and collaborative problem-solving. Students appreciated the opportunity to learn from peers in other disciplines and felt emotionally connected when working together on cases. The web-based format, with its structured checkpoints and group discussions, also contributed to a sense of belonging and engagement.

Intrinsic motivation was enhanced when students found the digital learning environment engaging and motivating, particularly due to its interactive elements, such as mini-games and autonomy in learning from the web-based platform and realistic simulations from IVR. These features supported self-directed learning, made the experience more enjoyable and meaningful, and relieved learning pressure.

Students reported that both the IVR and web-based platforms contributed to knowledge retention in learning, albeit in different ways, which was also supported by improved scores in MCQs that were related to IPE and were field-specific. Students reflected that the time constraints in IVR encouraged quick thinking and deeper memory encoding, while the web-based platform allowed for repeated practice and immediate feedback, reinforcing learning through repetition.

Students also reported some strengths and weaknesses in the focus groups. While students appreciated the innovative and engaging design of the learning experience, technical issues in both the IVR and web-based platforms, such as poor connectivity, disrupted the learning flow and experience. With regard to teaching content for future improvement, students suggested expanding the interprofessional scope to include more health care roles and enhancing the realism of simulations by incorporating patient interactions.

IVR provided an interesting and innovative experience but limited participation due to time constraints, whereas the web-based game offered enjoyment through mini-games and interactive elements, although technical issues were a major obstacle. The high level of flexibility and self-paced learning offered by the web-based game, along with the immersive and realistic experience of IVR, led to high engagement in the IPE program. Students who preferred the web-based game as a learning platform appreciated its stronger interactive elements and rich knowledge content, while those who preferred IVR valued its immersive experience and smooth operation. This satisfaction was reflected in significantly higher BSCS and MCQ scores, as well as in reduced pressure/tension scores. Through the IPE program, students were able to improve their understanding of interprofessional roles as one of the learning outcomes. Both platforms facilitated deeper memory retention in learning, as evidenced by improved scores in MCQs that were related to IPE and were field-specific. The IVR helped to deepen memory with intensive time constraints, while the web-based game strengthened memory with repeated practice. However, students felt that the 2 platforms had no strong association for enhancing the learning experience. As an interprofessional collaboration training tool, students agreed that it offered a chance for interprofessional collaboration, but some students felt that collaboration in IVR was limited and that there were insufficient interdisciplinary tasks.

Technical issues were identified in both the IVR and web-based game, significantly affecting the user experience. With regard to IVR, some students experienced difficulties with VR controls, and the limited number of devices prevented simultaneous engagement from all group members. With regard to the web-based game, frequent disconnections and login issues were reported, and the limited number of participants allowed in rooms for group discussions hindered the smoothness of the learning process.

In terms of areas for improvement, students suggested enhancing technical support for both platforms, incorporating more interactive tasks, and increasing the duration of sessions for IVR to more than 2 hours. Additionally, for future development, students recommended including professionals from more disciplines in the IPE, such as doctors and social workers, and adding patient interaction in simulation scenarios.

### Harms

No adverse events were reported by students in the study.

## Discussion

### Principal Findings

To our knowledge, this is the first study to directly compare the efficacy of IVR and web-based serious games in fostering interprofessional competencies and learning experiences among health care students. Both approaches yielded notable improvements in community building, motivation, and knowledge acquisition, although no significant changes were observed in readiness for interprofessional teamwork. The web-based approach led to comparatively stronger gains in knowledge clarity and motivation, possibly due to its flexible and diversified learning formats that cater to different learning preferences so as to support knowledge retention. Overall, the findings suggest that both modalities hold practical value in advancing technology-enhanced IPE, with distinct strengths depending on learning context and objectives.

### Interpretation

Both groups demonstrated a clear and significant improvement in community building, as reflected by increased scores in the BSCS. Consistent with the quantitative findings, the focus group participants emphasized the importance of interdisciplinary learning, stating that collaborating with peers from different disciplines enriched their perspectives, deepened their understanding, and fostered emotional connection through shared case discussions [43]. The web-based platform’s structured checkpoints and group discussions particularly promoted engagement and a sense of belonging, while supportive communication encouraged participation across both modalities.

The existing literature supports these findings, showing that students who perceive themselves as part of a cohesive learning community are better prepared for interprofessional collaboration, a vital skill in delivering high-quality patient care [44]. A strong sense of community is also associated with improved academic performance, greater satisfaction with the learning experience, and better management of stress and anxiety—common challenges among health care students due to their rigorous coursework and examinations [45]. Therefore, cultivating a sense of community with their peers through IPE would not only enhance learning outcomes but also prepare students for the collaborative, team-based practice central to effective health care delivery [46]. This study revealed significant changes in IMI subscale scores among students using both the IVR and web-based learning platforms. The IMI was used to assess different dimensions of motivation related to learning approaches [42], including interest/enjoyment**,** perceived competence**,** and pressure/tension. Both groups demonstrated a statistically significant reduction in pressure and tension. These findings suggest lower anxiety levels and a more supportive learning atmosphere across both modalities [47].

Consistent with these quantitative findings, the focus group participants indicated that the variety of mini-games reduced fatigue and increased engagement, and they appreciated the authenticity of the simulated hospital environment, which enhanced the realism and perceived value of the experience. Together, these insights indicate that the reduced anxiety in both the IVR and web-based contexts can facilitate collaborative learning, as lower stress levels encourage active engagement and deeper comprehension, which are factors critical in health care education [48]. Reduced anxiety promotes active engagement and deeper learning comprehension, which are particularly vital in health care education, while enhancing intrinsic motivation [49].

The observed decrease in pressure/tension among IVR learners is consistent with prior research showing that immersive virtual environments can alleviate anxiety and stress, promoting a more relaxed and receptive learning state [50]. Similarly, a systematic review indicated that IVR significantly bolstered student motivation and engagement, culminating in improved learning outcomes [51]. Likewise, a study determined that the learning environments of IVR-based serious games promote motivation, engagement, and interaction, and enable personalized and collaborative learning [52]. When students establish connections with their peers and encounter reduced pressure, they are more inclined to engage actively in discussions, exchange resources, and collaborate efficiently on projects. This enhances their learning experience and aids in the development of crucial skills necessary for effective teamwork in clinical settings, which are essential for patient care in health care education [53].

Apart from a reduction in pressure, students in the web-based group also showed improvements in intrinsic motivation, as reflected by increased scores in the IMI subscales for interest/enjoyment and perceived competence, with narrow 95% CIs. Students expressed the view that the web-based platform allowed for more self-directed learning, providing opportunities for exploration and instant feedback, which are beneficial for their learning. This result is substantiated by a prior systematic review revealing that game-based learning is better than learning without gamification in improving students’ perceptions of autonomy, relatedness, and competence [54]. Another review indicated that game-based learning has positive effects on students’ motivation levels over the short term [55]. Research indicates that intrinsically motivated students are more likely to attain higher levels of academic performance [56].

While both groups demonstrated notable enhancements in learning outcomes, as reflected by an increase in their knowledge-checking MCQ scores after completing the IPE, the web-based group outperformed the IVR group, with a statistically significant and moderately sized advantage. This is consistent with prior research, which indicated that web-based learning typically improves engagement and enhances knowledge retention [57,58]. This difference may be attributed to the time limitations encountered by the IVR group, in contrast to the web-based group, which benefited from increased flexibility and the chance for self-directed learning, allowing for repeated practice and deeper comprehension [59]. A few students noted that the time limit in IVR forced them to think quickly, suggesting that this challenge might enhance memory retention and learning depth in some learners. However, some students pointed out that the web-based platform’s ability to allow repeated gameplay is akin to practicing the filling out of examination papers, which more effectively reinforces learning and memory retention. They remarked that in IVR, mistakes are acknowledged but participants quickly move on without deeper engagement, while the web-based format provides a more thorough learning experience. Such feedback indicates that a single approach may not be sufficient to meet the learning styles of all students, who have diverse preferences in terms of their education [60].

In our study, students who participated in IVR and web-based learning did not show improvements in terms of their preparedness for interprofessional collaboration, as measured by the RIPLS. This finding aligns with emerging evidence suggesting that sustained and longitudinal IPE may be necessary to achieve a meaningful impact on readiness for interprofessional collaboration. Research has demonstrated that while brief IPE programs may show limited immediate improvements in RIPLS scores, extended exposure to IPE is often required to detect significant changes [61]. Similarly, longitudinal studies have indicated that the development of interprofessional competency is a gradual process requiring sustained practice and reflection over extended periods [62,63]. Interprofessional collaboration skill development involves the acquisition of a range of competencies, including communication skills, conflict resolution abilities, and effective teamwork [32]. The development of these skills typically requires a substantial amount of time and sustained practice. The current IVR and web-based IPE activities, which were conducted over a period of approximately 1 week, may not have been sufficient to meet these standards [44]. Students may have had limited opportunities to engage in meaningful interactions, reflect on their experiences, and consolidate their learning. Therefore, students may not have attempted to internalize interprofessional teamwork concepts that were actively taught, thus highlighting insufficient pedagogical guidance [64]. Future studies should consider implementing longitudinal serious gaming interventions with sustained exposure over multiple months or academic terms to assess and develop readiness for interprofessional collaboration.

Web-based platforms have demonstrated comparatively greater advantages than IVR approaches in IPE, especially for health care professionals facing scheduling and geographical constraints [17]. They provide flexibility for self-paced learning, remove geographical barriers, and accommodate a larger number of participants, promoting inclusivity [65]. Furthermore, web-based learning supports various formats (eg, videos, quizzes, and interactive modules) that address different learning preferences, thereby increasing engagement [66]. Additionally, the cost-effectiveness of online education minimizes travel and accommodation costs [66]. Real-time collaboration tools also provide immediate feedback and discussion opportunities, enhancing the overall learning experience [65]. Lastly, the vast array of online resources enriches educational experience, making web-based platforms a more effective and inclusive option for IPE.

### Limitations

This study has several limitations. First, it included only undergraduate nursing and physiotherapy students, and thus, it may not have captured the full range of perspectives and dynamics present in real-world interprofessional teams. The exclusion of individuals from other disciplines, such as medicine, pharmacy, social work, occupational therapy, and speech-language pathology, may have limited insights into the broader dynamics of collaboration, clinical decision-making, medication management, psychosocial support, and specialized rehabilitation. Second, most outcomes were assessed using self-reported questionnaires, which may have introduced an expectation bias; however, anonymity was implemented to reduce this bias. Additionally, qualitative findings cannot be generalized to a broader population since they are often based on personal experiences rather than on quantifiable data. Third, technical issues, such as difficulties with VR controls and connectivity, likely disrupted learning continuity and negatively affected student perceptions. These disruptions may have impacted immersion and collaboration, confounding assessments of true educational impacts. Lastly, both IVR and web-based platforms lacked integrated patient interactions within simulation scenarios, which may have reduced the realism of the learning experience. Future studies should involve participants from a broader range of health care disciplines to enhance ecological validity; strengthen technical support and user training; and incorporate simulated patient encounters to improve authenticity, clinical reasoning, and engagement. Addressing these limitations will advance the development of technology-enhanced IPE and better prepare students for collaborative, real-world health care practice [67,68].

### Conclusion

To our knowledge, this is the first study to directly compare the effects of IVR and web-based serious games on an IPE program. It differs from existing studies by contrasting 2 widely used but rarely directly compared technology-enabled modalities within the same authentic clinical case flow. Our study combined quantitative and qualitative data to provide a more comprehensive understanding of how the educational tools of IVR and web-based serious games can improve the interprofessional skills of health care students. Both methods demonstrated notable advancements in community feeling and subject-specific knowledge; however, the web-based approach surpassed the IVR method in terms of knowledge clarity and participant engagement. Although both platforms led to decreased pressure or tension, neither method significantly enhanced readiness for interprofessional teamwork, indicating possible shortcomings in the design of activities and educational support. The results underscore the necessity for engaging and supportive learning environments that reduce anxiety/stress and boost motivation while also tackling technical issues to optimize educational outcomes and better equip students for collaborative roles in health care. In real-world practice, these findings suggest that educational institutions can strategically integrate a variety of serious game elements into IPE training to better prepare students for collaborative, patient-centered care.

## Supplementary material

10.2196/80033 Multimedia Appendix 1 Multiple choice questions developed and used to measure students’ learning outcomes.

10.2196/80033 Multimedia Appendix 2 Interview questions for the focus group.

10.2196/80033 Multimedia Appendix 3 Between-group comparisons of primary outcome measures.

10.2196/80033 Multimedia Appendix 4 Within-group comparisons of primary outcome measures.

10.2196/80033 Checklist 1 CONSORT checklist.

10.2196/80033 Checklist 2 CONSORT‐EHEALTH checklist.

## Abbreviations

BSCS: Brief Sense of Community Scale
CONSORT: Consolidated Standards of Reporting Trials
IMI: Intrinsic Motivation Inventory
IPE: interprofessional education
IVR: immersive virtual reality
MANOVA: multivariate analysis of variance
MCQs: multiple-choice questions
RIPLS: Readiness for Interprofessional Learning Scale
