# Supplementary material for: Exploring the Effects of Serious Games (Immersive Virtual Reality Versus Web-Based Platforms) on Interprofessional Education Among Undergraduate Health Care Students: Randomized Controlled Trial and Multimethod Study
Source: JMIR Serious Games. 2026 May 25;14:e80033. doi: 10.2196/80033 (PMC13200777; doi:10.2196/80033)
Supplement: Multimedia Appendix 1 [file games-v14-e80033-s001.docx]

**Multimedia Appendix 1**

**Multiple choice questions developed and used to measure students’ learning outcomes**

Q1. How long is the typical time duration for the Chair Stand Test?

- a) 10 seconds (1)
- b) 30 seconds (2)
- c) 1 minute (3)
- d) 2 minutes (4)

Q2 In the Time Up and Go (TUG) test, what is the standard distance a person walks before turning around?

- a) 1 meter (1)
- b) 2 meters (2)
- c) 3 meters (3)
- d) 4 meters (4)

Q3 What is considered a normal or average time for healthy adults to complete the TUG test?

- a) Less than 5 seconds (1)
- b) 10 to 20 seconds (2)
- c) 30 to 40 seconds (3)
- d) More than 1 minute (4)

Q4 Which of the following is a common cutoff score for SARC-F to indicate a high risk of sarcopenia?

- a) 1 (1)
- b) 2 (2)
- c) 3 (3)
- d) 4 (4)

Q5 Which of the following is NOT a component of the SARC-F questionnaire?

- a) Strength (1)
- b) Activity level (2)
- c) Resilience (3)
- d) Walking ability (4)

Q6 Which of the following is not an element of an intervention program in a hospital setting

- A. Staff education on fall precautions (1)
- B. Provision and maintenance of mobility aids (2)
- C. Post fall analysis and problem-solving strategy (3)
- D. Bed alarms for all patients, regardless of risk (4)

Q7 Risk factors for falls do not include

- A. Body weight (1)
- B. Incontinence (2)
- C. Previous history of falls (3)
- D. Delirium (4)

Q8 Exercise programs for ambulatory older adults should

- A. Be very aggressive (1)
- B. Be unsupervised (2)
- C. Have the objective of attaining accelerated muscle development outcomes (3)
- D. Include individualized strength and balance training (4)

Q9 Which of the following statements on education in fall prevention is false?

- A. Education programs should target primarily healthcare providers, patients, and caregivers. (1)
- B. Education programs for staff should include the importance of fall prevention, risk factors for falls, strategies to reduce falls, and transfer techniques. (2)
- C. Education should only be given at the start of the fall prevention program. (3)
- D. Instruction on safe mobility, with emphasis on high-risk patients, should be provided to both patients and families. (4)

Q10 Which of the following is not recommended to improve patient safety?

- A. Locking wheeled furniture when it is stationary. (1)
- B. Having nonslip flooring. (2)
- C. Placing frequently used items (including a call bell, telephone, and remote control) within reach of the patient. (3)
- D. Rounding once per day and having the call bells available for patients to use outside of designated times. (4)

Q11. Which of the following is a major risk factor for falls among the elderly?

- a) Being male (1)
- b) Having decreased muscle strength (5)
- c) Living with family (6)
- d) Having an active lifestyle (7)

Q12 What kinds of medical conditions will NOT significantly increase the fall risk among the elderly?

- a) Stroke (1)
- b) Dementia (4)
- c) Pneumonia (5)
- d) Depression (6)

Q13 What is the most common environmental/external cause of falls among the elderly?

- a) Slippery floors (1)
- b) Improper footwear (4)
- c) Dim lighting (5)
- d) All of the above (6)

Q14 Which of the following can help reduce the risk of falls among the elderly?

- a) Wearing high-heeled shoes (1)
- b) Avoiding regular exercise (4)
- c) Removing handrails from the bathroom (5)
- d) Regularly reviewing medications (6)

Q15 If the patient has a fall history, it is important to record the following information to formulate individualized fall prevention education, EXCEPT for

- a) Time of falls (1)
- b) Location of falls (4)
- c) Activities during the fall (5)
- d) Fall mechanism (6)
- e) Whether any relatives were around during the fall (7)
- f) If there were any injuries or fractures after the fall (8)

Q16. Why is interprofessional collaboration important?

- a. Good work can be done in one clinical visit if there is a strong alliance among the healthcare team (1)
- b. The administrators at the organizational level can implement their vision effectively (4)
- c. Team members must monitor each other’s performance in order to ensure a high quality of care (5)
- d. One professional can take up the role of another member in the team to ensure integrated care (6)

Q17. Healthcare professionals can gain advantages from working as an interprofessional team rather than working in silo. One of these advantages is:

- a. Better adherence to conventional strategies in managing health (3)
- b. More proficiency in profession-specific knowledge and skills (4)
- c. Improved efficiency and responsiveness to patient-centered care (5)
- d. Better protection of the resources for advancing profession-specific development (6)

Q18. An interprofessional collaborative culture is reflected in one of the following statements:

- a. Maximizing the benefits of the profession while working as an interprofessional team (3)
- b. Applying the principles of team dynamics to promptly deliver health programs (4)
- c. Regularly accrediting the existing professional qualifications (5)
- d. Ensuring that research results on the needs of the community are published (6)

Q19. Health interprofessional dynamics thrive on:

- a. Productive dialogues for innovative solutions (1)
- b. Enforcing conformity and consensus in decision making (4)
- c. Continuous collaborative research findings (5)
- d. Creative interventions to meet patients’ preferences (6)

Q20. The foundation to build an optimal collaborative work culture relies on:

- a. A well-established error-free work environment and risk management (1)
- b. Well-established governance models and structured protocols (4)
- c. Institutional supports to create synergies and shape practices among the healthcare team (5)
- d. New skills available to resolve complex problems across the healthcare team (6)
