# Supplementary material for: Exploring the Effects of Serious Games (Immersive Virtual Reality Versus Web-Based Platforms) on Interprofessional Education Among Undergraduate Health Care Students: Randomized Controlled Trial and Multimethod Study
Source: JMIR Serious Games. 2026 May 25;14:e80033. doi: 10.2196/80033 (PMC13200777; doi:10.2196/80033)
Supplement: Multimedia Appendix 2 [file games-v14-e80033-s002.docx]

**Multimedia Appendix 2**

**Interview questions for the focus group**

# Interprofessional Collaboration

1. Teamwork: How did the training programs impact your ability to work in an interprofessional team?
2. Communication: Did you notice any changes in your communication skills with team members from different disciplines?

# Sense of Community

1. Overall Experience: Can you describe your overall experience with the iHealthEd-IVR and web-based training programs?
2. Engagement: How engaging did you find the training sessions? What aspects kept you most engaged?

# Intrinsic Motivation

1. Motivation: How did the training programs affect your motivation to learn and participate in interprofessional education?

# Learning Outcomes

1. Knowledge and Skills: What specific knowledge or skills did you gain from participating in these programs?
2. Application: Can you share how you will apply what you learned from the IPE training in the future?

# Strengths and Weaknesses

1. Technology and Tools
   1. iHealthEd-IVR: What was your experience using the immersive VR technology? Were there any challenges or benefits?
   2. Web-based game: What was your experience using the mobile game platform for learning? What features stood out to you?
2. Program and Content
   1. Effectiveness: Between the iHealthEd-IVR and the web-based game, which method did you find more effective for your learning and why?
   2. Preference: If you had to choose, which training method would you prefer for future IPE activities?

# Improvements and Suggestions

1. Challenges: What challenges did you face during the training programs?
2. Suggestions: Do you have any suggestions for improving the training programs?
3. Future Directions: What other topics or areas would you like to see covered in future IPE training programs?
