# Supplementary material for: Exploring the Effects of Serious Games (Immersive Virtual Reality Versus Web-Based Platforms) on Interprofessional Education Among Undergraduate Health Care Students: Randomized Controlled Trial and Multimethod Study
Source: JMIR Serious Games. 2026 May 25;14:e80033. doi: 10.2196/80033 (PMC13200777; doi:10.2196/80033)
Supplement: Multimedia Appendix 3 [file games-v14-e80033-s003.docx]

**Multimedia Appendix 3.** Between-group comparisons of primary outcome measures.

|  | Pre | | 95% Confidence Interval | Effect Size  *r*^b^ | z score  *z* | *P*-value^a^ | Post | | 95% Confidence Interval | Effect Size  *r*^b^ | z score  *z* | *P*-value^a^ |
| --- | --- | --- | --- | --- | --- | --- | --- | --- | --- | --- | --- | --- |
| *Outcome Measures* | IVR Group Median (IQR) | V-Care Group Median (IQR) |  |  |  |  | IVR Group Median (IQR) | V-Care Group Median (IQR) |  |  |  |  |
| Readiness for Interprofessional Learning Scale (RIPLS) *(score ↑, more positive attitude*) | | | | | | | | | | | | |
| Overall | 3.58 (3.42-3.84) | 3.63 (3.47-3.86) | [-0.11,0.05] | -0.06 | -1.01 | .314 | 3.58 (3.37-3.79) | 3.63 (3.42-3.89) | [-0.16,0.00] | -0.10 | -1.56 | .110 |
| Teamwork and Collaboration | 4.00 (3.89-4.33) | 4.00 (3.89-4.33) | [-0.11,0.11] | -0.01 | -0.23 | .822 | 4.00 (3.78-4.28) | 4.00 (4.00-4.22) | [-0.11,0.00] | -0.04 | -0.71 | .480 |
| Professional Identity | 3.29 (3.14-3.43) | 3.29 (3.14-3.46) | [-0.14,0.00] | -0.07 | -1.11 | .268 | 3.14 (3.00-3.43) | 3.29 (3.14-3.57) | [-0.14,0.00] | -0.11 | -1.77 | .076 |
| Roles and Responsibility | 3.00 (2.67-3.33) | 3.00 (2.92-3.33) | [-0.33,0.00] | -0.07 | -1.15 | .250 | 3.00 (2.67-3.33) | 3.00 (2.67-3.67) | [-0.33,0.00] | -0.10 | -1.60 | .109 |
| Brief Sense of Community Scale (BSCS) *(score ↑, stronger sense of community*) | | | | | | | | | | | | |
| Overall | 3.38 (3.00-3.88) | 3.44 (3.00-4.00) | [-0.13,0.00] | -0.07 | -1.1 | .272 | 3.63 (3.19-4.00) | 3.75 (3.38-4.00) | [-0.25,0.00] | -0.09 | -1.48 | .140 |
| Needs Fulfillment | 3.00 (3.00-4.00) | 3.50 (3.00-4.00) | [0.00,0.00] | -0.04 | -0.71 | .476 | 4.00 (3.00-3.50) | 4.00 (3.00-4.00) | [0.00,0.00] | -0.12 | **-2.05** | **.040** |
| Membership | 3.00 (3.00-3.50) | 3.50 (3.00-4.00) | [0.00,0.00] | -0.12 | **-2.02** | **.044** | 3.50 (3.00-4.00) | 3.50 (3.00-4.00) | [-0.50,0.00] | -0.13 | **-2.09** | **.037** |
| Influence | 3.00 (3.00-4.00) | 3.50 (3.00-4.00) | [0.00,0.00] | -0.02 | -0.34 | .737 | 4.00 (3.00-4.00) | 4.00 (3.00-4.00) | [0.00,0.00] | -0.02 | -0.26 | .794 |
| Emotional Connection | 3.00 (3.00-4.00) | 3.50 (3.00-4.00) | [0.00,0.00] | -0.07 | -1.12 | .225 | 4.00 (3.50-4.00) | 4.00 (3.00-4.00) | [0.00,0.00] | -0.01 | -0.12 | .902 |
| Intrinsic Motivation Inventory (IMI) *(score ↑, motivation and experience ↑*) | | | | | | | | | | | | |
| Interest/ Enjoyment | 4.00 (4.00-4.60) | 4.20 (4.00-4.56) | [0.00,0.00] | -0.03 | -0.50 | .615 | 4.20 (3.80-5.00) | 4.60 (4.00-5.25) | [-0.20,-0.40] | -0.09 | -1.43 | .154 |
| Perceived Competence | 4.00 (4.00-4.50) | 4.00 (4.00-4.50) | [0.00,0.00] | -0.08 | -1.26 | .208 | 4.00 (3.50-5.00) | 4.00 (4.00-5.00) | [0.00,-0.50] | -0.06 | -1.03 | .304 |
| Pressure/ Tension | 4.00 (3.50-4.00) | 4.00 (3.88-4.00) | [0.00,0.00] | -0.13 | **-2.14** | **.033** | 3.50 (2.50-4.00) | 3.50 (3.00-4.00) | [0.00,0.00] | -0.08 | -0.32 | .749 |
| Multiple choice questions (MCQs) *(score ↑, clearer knowledge*) | | | | | | | | | | | | |
| Total | 13.00 (9.00-15.00) | 12.00 (8.00-14.00) | [1.00,0.00] | -0.09 | -1.42 | 0.157 | 15.00 (13.00-17.00) | 18.00 (15.00-19.00) | [-2.00,-3.00] | **-0.34** | **-5.53** | **<0.001** |

RIPLS=The Readiness for Interprofessional Learning Scale; BSCS=The Brief Sense of Community Scale; IMI= The Intrinsic Motivation Inventory questionnaire; MCQs=Multiple-choice questions;

a Mann-Whitney U Test; The bold value indicates statistically significant difference

b Effect size = z/√n
