# Supplementary material for: Exploring the Effects of Serious Games (Immersive Virtual Reality Versus Web-Based Platforms) on Interprofessional Education Among Undergraduate Health Care Students: Randomized Controlled Trial and Multimethod Study
Source: JMIR Serious Games. 2026 May 25;14:e80033. doi: 10.2196/80033 (PMC13200777; doi:10.2196/80033)
Supplement: Multimedia Appendix 4 [file games-v14-e80033-s004.docx]

**Multimedia Appendix 4.** Within-group comparisons of primary outcome measures.

|  | IVR Group (n=125) | | 95% Confidence Interval | Effect Size  *r*^b^ | z score  *z* | *P*-value^a^ | Web-based Group (n=146) | | 95% Confidence Interval | Effect Size  *r*^b^ | z score  *z* | *P*-value^a^ |
| --- | --- | --- | --- | --- | --- | --- | --- | --- | --- | --- | --- | --- |
| *Outcome Measures* | Pre Median (IQR) | Post Median (IQR) |  |  |  |  | Pre Median (IQR) | Post Median (IQR) |  |  |  |  |
| Readiness for Interprofessional Learning Scale (RIPLS) *(score ↑, more positive attitude*) | | | | | | | | | | | | |
| Total | 3.58 (3.42-3.84) | 3.58 (3.37-3.79) | [-0.11,0.03] | -0.10 | -1.17 | .242 | 3.63 (3.47-3.86) | 3.63 (3.42-3.89) | [-0.05,0.05] | -0.01 | -0.13 | .895 |
| Teamwork and Collaboration | 4.00 (3.89-4.33) | 4.00 (3.78-4.28) | [-0.17,0.00] | -0.14 | -1.57 | .116 | 4.00 (3.89-4.33) | 4.00 (4.00-4.22) | [-0.06,0.06] | -0.03 | -0.32 | .749 |
| Professional Identity | 3.29 (3.14-3.43) | 3.14 (3.00-3.43) | [-0.07,0.07] | -0.07 | -0.73 | .463 | 3.29 (3.14-3.46) | 3.29 (3.14-3.57) | [-0.07,0.07] | -0.01 | -0.16 | .873 |
| Roles and Responsibility | 3.00 (2.67-3.33) | 3.00 (2.67-3.33) | [-0.17,0.00] | -0.03 | -0.30 | .768 | 3.00 (2.92-3.33) | 3.00 (2.67-3.67) | [-0.00,0.17] | -0.02 | -0.28 | .782 |
| Brief Sense of Community Scale (BSCS) *(score ↑, stronger sense of community*) | | | | | | | | | | | | |
| Total | 3.38 (3.00-3.88) | 3.63 (3.19-4.00) | [0.06,0.31] | -0.29 | **-3.21** | **.001** | 3.44 (3.00-4.00) | 3.75 (3.38-4.00) | [0.07,0.31] | -0.27 | **-3.27** | **.001** |
| Needs Fulfillment | 3.00 (3.00-4.00) | 4.00 (3.00-3.50) | [0.00,0.25] | -0.10 | -1.08 | .279 | 3.50 (3.00-4.00) | 4.00 (3.00-4.00) | [0.00,0.50] | -0.18 | **-2.18** | **.030** |
| Membership | 3.00 (3.00-3.50) | 3.50 (3.00-4.00) | [0.00,0.25] | -0.13 | -1.40 | .162 | 3.50 (3.00-4.00) | 3.50 (3.00-4.00) | [0.00,0.25] | -0.11 | -1.33 | .183 |
| Influence | 3.00 (3.00-4.00) | 4.00 (3.00-4.00) | [0.00,0.50] | **-0.32** | **-3.72** | **<.001** | 3.50 (3.00-4.00) | 4.00 (3.00-4.00) | [0.00,0.50] | **-0.30** | **-.365** | **<.001** |
| Emotional Connection | 3.00 (3.00-4.00) | 4.00 (3.50-4.00) | [0.25,0.50] | **-0.33** | **-3.21** | **<.001** | 3.50 (3.00-4.00) | 4.00 (3.00-4.00) | [0.00,0.50] | -0.24 | **-2.85** | **.004** |
| Intrinsic Motivation Inventory (IMI) *(score ↑, motivation and experience ↑*) | | | | | | | | | | | | |
| Interest/ Enjoyment | 4.00 (4.00-4.60) | 4.20 (3.80-5.00) | [-0.10,0.20] | -0.05 | -0.57 | .571 | 4.20 (4.00-4.56) | 4.60 (4.00-5.25) | [0.00,0.30] | -0.17 | **-2.03** | **.043** |
| Perceived Competence | 4.00 (4.00-4.50) | 4.00 (3.50-5.00) | [0.00,0.25] | -0.17 | -5.07 | .054 | 4.00 (4.00-4.50) | 4.00 (4.00-5.00) | [0.00,0.50] | -0.19 | **-2.29** | **.022** |
| Pressure/ Tension | 4.00 (3.50-4.00) | 3.50 (2.50-4.00) | [-0.50,-0.25] | **-0.45** | **-0.41** | **<.001** | 4.00 (3.88-4.00) | 3.50 (3.00-4.00) | [-0.75,-0.50] | **-0.46** | **-5.58** | **<.001** |
| Multiple choice questions (MCQs) *(score ↑, clearer knowledge*) | | | | | | | | | | | | |
| Total | 13.00 (9.00-15.00) | 15.00 (13.00-17.00) | [2.00,3.00] | **-0.64** | **-7.75** | **<.001** | 12.00 (8.00-14.00) | 18.00 (15.00-19.00) | [4.50,6.00] | **-0.80** | **-9.63** | **<.001** |

RIPLS=The Readiness for Interprofessional Learning Scale; BSCS=The Brief Sense of Community Scale; IMI= The Intrinsic Motivation Inventory questionnaire; MCQs=Multiple-choice questions

a Wilcoxon Signed Rank Test; The bold value indicates statistically significant difference.

b Effect size = z/√n
