# Supplementary material for: Exploring the Effects of Serious Games (Immersive Virtual Reality Versus Web-Based Platforms) on Interprofessional Education Among Undergraduate Health Care Students: Randomized Controlled Trial and Multimethod Study
Source: JMIR Serious Games. 2026 May 25;14:e80033. doi: 10.2196/80033 (PMC13200777; doi:10.2196/80033)
Supplement: Checklist 2 [file games-v14-e80033-s006.pdf]

CONSORT-EHEALTH (V 1.6.1) - Submission/Publication Form

The CONSORT-EHEALTH checklist is intended for authors of randomized trials evaluating web-based and Internet-based applications/interventions, including mobile interventions, electronic games (incl multiplayer games), social media, certain telehealth applications, and other interactive and/or networked electronic applications. Some of the items (e.g. all subitems under item 5 - description of the intervention) may also be applicable for other study designs.

The goal of the CONSORT EHEALTH checklist and guideline is to be
a) a guide for reporting for authors of RCTs,
b) to form a basis for appraisal of an ehealth trial (in terms of validity)

CONSORT-EHEALTH items/subitems are MANDATORY reporting items for studies published in the Journal of Medical Internet Research and other journals / scientific societies endorsing the checklist.

Items numbered 1., 2., 3., 4a., 4b etc are original CONSORT or CONSORT-NPT (non-pharmacologic treatment) items.
Items with Roman numerals (i., ii, iii, iv etc.) are CONSORT-EHEALTH extensions/clarifications.

As the CONSORT-EHEALTH checklist is still considered in a formative stage, we would ask that you also RATE ON A SCALE OF 1-5 how important/useful you feel each item is FOR THE PURPOSE OF THE CHECKLIST and reporting guideline (optional).

Mandatory reporting items are marked with a red *.
In the textboxes, either copy & paste the relevant sections from your manuscript into this form - please include any quotes from your manuscript in QUOTATION MARKS,
or answer directly by providing additional information not in the manuscript, or elaborating on why the item was not relevant for this study.

YOUR ANSWERS WILL BE PUBLISHED AS A SUPPLEMENTARY FILE TO YOUR PUBLICATION IN JMIR AND ARE CONSIDERED PART OF YOUR PUBLICATION (IF ACCEPTED).
Please fill in these questions diligently. Information will not be copyedited, so please use proper spelling and grammar, use correct capitalization, and avoid abbreviations.

DO NOT FORGET TO SAVE AS PDF _AND_ CLICK THE SUBMIT BUTTON SO YOUR ANSWERS ARE IN OUR DATABASE !!!

Citation Suggestion (if you append the pdf as Appendix we suggest to cite this paper in the caption):
Eysenbach G, CONSORT-EHEALTH Group

CONSORT-EHEALTH: Improving and Standardizing Evaluation Reports of Web-based and Mobile Health Interventions
J Med Internet Res 2011;13(4):e126
URL: http://www.jmir.org/2011/4/e126/
doi: 10.2196/jmir.1923
PMID: 22209829

wing-yan-rina.wong@polyu.edu.hk Switch account

Not shared

Draft saved

* Indicates required question

Your name *

First Last

Justina Yat Wa Liu

Primary Affiliation (short), City, Country *

University of Toronto, Toronto, Canada

School of Nursing, The Hong Kong Polytechnic

Your e-mail address *

abc@gmail.com

justina.liu@polyu.edu.hk

Title of your manuscript *

Provide the (draft) title of your manuscript.

Exploring the Effects of Serious Games Using Immersive Virtual Reality (IVR) versus Web-based Platforms on Interprofessional Education (IPE) for Undergraduate Healthcare Students: A Randomized Controlled Trial and Multiple-Methods Study

Name of your App/Software/Intervention *

If there is a short and a long/alternate name, write the short name first and add the long name in brackets.

(1) VR Hospital (Virtual Reality Hospital); (2) v-

Evaluated Version (if any)

e.g. "V1", "Release 2017-03-01", "Version 2.0.27913"

Your answer

Language(s) *

What language is the intervention/app in? If multiple languages are available, separate by comma (e.g. "English, French")

English

URL of your Intervention Website or App

e.g. a direct link to the mobile app on app in appstore (itunes, Google Play), or URL of the website. If the intervention is a DVD or hardware, you can also link to an Amazon page.

Your answer

URL of an image/screenshot (optional)

Your answer

Accessibility *

Can an enduser access the intervention presently?

- ( ) access is free and open
- (x) access only for special usergroups, not open
- ( ) access is open to everyone, but requires payment/subscription/in-app purchases
- ( ) app/intervention no longer accessible
- ( ) Other:

Primary Medical Indication/Disease/Condition *

e.g. "Stress", "Diabetes", or define the target group in brackets after the condition, e.g. "Autism (Parents of children with)", "Alzheimers (Informal Caregivers of)"

Interprofessional education (IPE) in healthcare

Primary Outcomes measured in trial *

comma-separated list of primary outcomes reported in the trial

Readiness for Interprofessional Learning Scale

Secondary/other outcomes

Are there any other outcomes the intervention is expected to affect?

Your answer

Recommended "Dose" *

What do the instructions for users say on how often the app should be used?

- ( ) Approximately Daily
- ( ) Approximately Weekly
- ( ) Approximately Monthly
- ( ) Approximately Yearly
- ( ) "as needed"
- (•) Other: according to the academic training program timeline

Approx. Percentage of Users (starters) still using the app as recommended after 3 months *

- (•) unknown / not evaluated
- ( ) 0-10%
- ( ) 11-20%
- ( ) 21-30%
- ( ) 31-40%
- ( ) 41-50%
- ( ) 51-60%
- ( ) 61-70%
- ( ) 71%-80%
- ( ) 81-90%
- ( ) 91-100%
- ( ) Other:

Overall, was the app/intervention effective? *

- ( ) yes: all primary outcomes were significantly better in intervention group vs control
- (•) partly: SOME primary outcomes were significantly better in intervention group vs control
- ( ) no statistically significant difference between control and intervention
- ( ) potentially harmful: control was significantly better than intervention in one or more outcomes
- ( ) inconclusive: more research is needed
- ( ) Other:

Article Preparation Status/Stage *

At which stage in your article preparation are you currently (at the time you fill in this form)

- ( ) not submitted yet - in early draft status
- ( ) not submitted yet - in late draft status, just before submission
- ( ) submitted to a journal but not reviewed yet
- (•) submitted to a journal and after receiving initial reviewer comments
- ( ) submitted to a journal and accepted, but not published yet
- ( ) published
- ( ) Other:

Journal *

If you already know where you will submit this paper (or if it is already submitted), please provide the journal name (if it is not JMIR, provide the journal name under "other")

- ( ) not submitted yet / unclear where I will submit this
- ( ) Journal of Medical Internet Research (JMIR)
- ( ) JMIR mHealth and UHealth
- (•) JMIR Serious Games
- ( ) JMIR Mental Health
- ( ) JMIR Public Health
- ( ) JMIR Formative Research
- ( ) Other JMIR sister journal
- ( ) Other:

Is this a full powered effectiveness trial or a pilot/feasibility trial? *

- ○ Pilot/feasibility
- ◉ Fully powered

Manuscript tracking number *

If this is a JMIR submission, please provide the manuscript tracking number under "other" (The ms tracking number can be found in the submission acknowledgement email, or when you login as author in JMIR. If the paper is already published in JMIR, then the ms tracking number is the four-digit number at the end of the DOI, to be found at the bottom of each published article in JMIR)

- ○ no ms number (yet) / not (yet) submitted to / published in JMIR
- ◉ Other: #80033

TITLE AND ABSTRACT

1a) TITLE: Identification as a randomized trial in the title

1a) Does your paper address CONSORT item 1a? *

I.e does the title contain the phrase "Randomized Controlled Trial"? (if not, explain the reason under "other")

- ◉ yes
- ○ Other:

1a-i) Identify the mode of delivery in the title

Identify the mode of delivery. Preferably use “web-based” and/or “mobile” and/or “electronic game” in the title. Avoid ambiguous terms like “online”, “virtual”, “interactive”. Use “Internet-based” only if Intervention includes non-web-based Internet components (e.g. email), use “computer-based” or “electronic” only if offline products are used. Use “virtual” only in the context of “virtual reality” (3-D worlds). Use “online” only in the context of “online support groups”. Complement or substitute product names with broader terms for the class of products (such as “mobile” or “smart phone” instead of “iphone”), especially if the application runs on different platforms.

	1	2	3	4	5	
subitem not at all important	○	○	○	○	◉	essential

Clear selection

Does your paper address subitem 1a-i? *

Copy and paste relevant sections from manuscript title (include quotes in quotation marks "like this" to indicate direct quotes from your manuscript), or elaborate on this item by providing additional information not in the ms, or briefly explain why the item is not applicable/relevant for your study

"Exploring the Effects of Serious Games Using Immersive Virtual Reality (IVR) versus Web-based Platforms on Interprofessional Education (IPE) for Undergraduate Healthcare Students: A Randomized Controlled Trial and Multiple-Methods Study"

1a-ii) Non-web-based components or important co-interventions in title

Mention non-web-based components or important co-interventions in title, if any (e.g., “with telephone support”).

	1	2	3	4	5	
subitem not at all important	○	○	○	○	○	essential

Does your paper address subitem 1a-ii?

Copy and paste relevant sections from manuscript title (include quotes in quotation marks "like this" to indicate direct quotes from your manuscript), or elaborate on this item by providing additional information not in the ms, or briefly explain why the item is not applicable/relevant for your study

Your answer

1a-iii) Primary condition or target group in the title

Mention primary condition or target group in the title, if any (e.g., "for children with Type I Diabetes") Example: A Web-based and Mobile Intervention with Telephone Support for Children with Type I Diabetes: Randomized Controlled Trial

	1	2	3	4	5	
subitem not at all important	○	○	○	○	◉	essential

Clear selection

Does your paper address subitem 1a-iii? *

Copy and paste relevant sections from manuscript title (include quotes in quotation marks "like this" to indicate direct quotes from your manuscript), or elaborate on this item by providing additional information not in the ms, or briefly explain why the item is not applicable/relevant for your study

"Interprofessional Education (IPE) in Undergraduate Healthcare Students"

1b) ABSTRACT: Structured summary of trial design, methods, results, and conclusions

NPT extension: Description of experimental treatment, comparator, care providers, centers, and blinding status.

1b-i) Key features/functionalities/components of the intervention and comparator in the METHODS section of the ABSTRACT

Mention key features/functionalities/components of the intervention and comparator in the abstract. If possible, also mention theories and principles used for designing the site. Keep in mind the needs of systematic reviewers and indexers by including important synonyms. (Note: Only report in the abstract what the main paper is reporting. If this information is missing from the main body of text, consider adding it)

	1	2	3	4	5	
subitem not at all important	○	○	○	○	○	essential

Does your paper address subitem 1b-i? *

Copy and paste relevant sections from the manuscript abstract (include quotes in quotation marks "like this" to indicate direct quotes from your manuscript), or elaborate on this item by providing additional information not in the ms, or briefly explain why the item is not applicable/relevant for your study

The study main focus is to identify an effective method for implementing IPE by assessing the impact of IVR and web-based serious games on healthcare students' interprofessional competencies and learning experiences. The "Methods" section of the abstract mainly focus on how to evaluate the primary outcomes on interprofessional competencies and learning experiences. Hence, we only mentioned the key features, functionalities, components of the intervention detailly in the main text.

1b-ii) Level of human involvement in the METHODS section of the ABSTRACT

Clarify the level of human involvement in the abstract, e.g., use phrases like “fully automated” vs. “therapist/nurse/care provider/physician-assisted” (mention number and expertise of providers involved, if any). (Note: Only report in the abstract what the main paper is reporting. If this information is missing from the main body of text, consider adding it)

	1	2	3	4	5	
subitem not at all important	○	○	○	○	◉	essential

Clear selection

Does your paper address subitem 1b-ii?

Copy and paste relevant sections from the manuscript abstract (include quotes in quotation marks "like this" to indicate direct quotes from your manuscript), or elaborate on this item by providing additional information not in the ms, or briefly explain why the item is not applicable/relevant for your study

"Nursing and physiotherapy students were randomly assigned to either the IVR group or web-based group. "

1b-iii) Open vs. closed, web-based (self-assessment) vs. face-to-face assessments in the METHODS section of the ABSTRACT

Mention how participants were recruited (online vs. offline), e.g., from an open access website or from a clinic or a closed online user group (closed usergroup trial), and clarify if this was a purely web-based trial, or there were face-to-face components (as part of the intervention or for assessment). Clearly say if outcomes were self-assessed through questionnaires (as common in web-based trials). Note: In traditional offline trials, an open trial (open-label trial) is a type of clinical trial in which both the researchers and participants know which treatment is being administered. To avoid confusion, use “blinded” or “unblinded” to indicated the level of blinding instead of “open”, as “open” in web-based trials usually refers to “open access” (i.e. participants can self-enrol). (Note: Only report in the abstract what the main paper is reporting. If this information is missing from the main body of text, consider adding it)

	1	2	3	4	5	
subitem not at all important	○	○	○	○	○	essential

Does your paper address subitem 1b-iii?

Copy and paste relevant sections from the manuscript abstract (include quotes in quotation marks "like this" to indicate direct quotes from your manuscript), or elaborate on this item by providing additional information not in the ms, or briefly explain why the item is not applicable/relevant for your study

"randomly assigned to either the IVR group or web-based group. " which 2 groups are web-based.
Surveys (Readiness for Interprofessional Learning Scale (RIPLS), Brief Sense of Community Scale (BSCS), and the Intrinsic Motivation Inventory (IMI)) were administered to assess learning experiences, while learning outcomes were examined through multiple-choice questions (MCQs)." has indicated that all outcome measures are self-assessed through questionnaires.

1b-iv) RESULTS section in abstract must contain use data

Report number of participants enrolled/assessed in each group, the use/uptake of the intervention (e.g., attrition/adherence metrics, use over time, number of logins etc.), in addition to primary/secondary outcomes. (Note: Only report in the abstract what the main paper is reporting. If this information is missing from the main body of text, consider adding it)

	1	2	3	4	5	
subitem not at all important	○	○	○	○	◉	essential

Clear selection

Does your paper address subitem 1b-iv?

Copy and paste relevant sections from the manuscript abstract (include quotes in quotation marks "like this" to indicate direct quotes from your manuscript), or elaborate on this item by providing additional information not in the ms, or briefly explain why the item is not applicable/relevant for your study

"A total of 271 students (125 in the IVR group and 146 in the web-based group) participated. Both groups significantly improved in community building (BSCS: IVR r=-0.29, 95% CI 0.06,0.31; web-based r=-0.27, 95% CI 0.07 to 0.31). For motivation, the IVR group improved only in the IMI subscales: “Pressure/Tension” (r=-0.45, 95% CI -0.50 to -0.25), while the web-based group improved across all IMI subscales: “interest/enjoyment” (r=-0.17, 95% CI 0.00 to 0.30), “perceived competence” (r=-0.19, 95% CI 0.00 to 0.50), and “pressure/tension” (r=-0.46, 95% CI -0.70 to -0.50). Both groups significantly improved MCQs knowledge scores (IVR: r=-0.64, 95% CI 2.00 to 3.00 and web-based scores: r=-0.80, 95% CI 4.50 to 6.00), with the web-based group outperforming the IVR group (r=-0.34, 95% CI -3.00 to -2.00). RIPLS scores showed no significant difference in both groups suggesting greater exposure is required to enhance IPE readiness. "
We have reported number of participants enrolled/assessed in each group and reporting the findings of each primary outcomes.

1b-v) CONCLUSIONS/DISCUSSION in abstract for negative trials

Conclusions/Discussions in abstract for negative trials: Discuss the primary outcome - if the trial is negative (primary outcome not changed), and the intervention was not used, discuss whether negative results are attributable to lack of uptake and discuss reasons. (Note: Only report in the abstract what the main paper is reporting. If this information is missing from the main body of text, consider adding it)

	1	2	3	4	5	
subitem not at all important	○	○	○	○	◉	essential

Clear selection

Does your paper address subitem 1b-v?

Copy and paste relevant sections from the manuscript abstract (include quotes in quotation marks "like this" to indicate direct quotes from your manuscript), or elaborate on this item by providing additional information not in the ms, or briefly explain why the item is not applicable/relevant for your study

To our knowledge, this is the first study to compare the effects of two different modalities (IVR and web-based) on a technology-enabled IPE program. These findings highlight the practical potential of both approaches in supporting IPE. The web-based approach's superior performance in enhancing learning outcomes and motivation suggests its flexible formats better accommodate diverse learning preferences. These findings support technology-enhanced IPE, offering educators scalable, evidence-based strategies to cultivate collaborative, patient-centered professionals equipped for real-world practice.

INTRODUCTION

2a) In INTRODUCTION: Scientific background and explanation of rationale

2a-i) Problem and the type of system/solution

Describe the problem and the type of system/solution that is object of the study: intended as stand-alone intervention vs. incorporated in broader health care program? Intended for a particular patient population? Goals of the intervention, e.g., being more cost-effective to other interventions, replace or complement other solutions? (Note: Details about the intervention are provided in “Methods” under 5)

	1	2	3	4	5	
subitem not at all important	○	○	○	○	◉	essential

Clear selection

Does your paper address subitem 2a-i? *

Copy and paste relevant sections from the manuscript (include quotes in quotation marks "like this" to indicate direct quotes from your manuscript), or elaborate on this item by providing additional information not in the ms, or briefly explain why the item is not applicable/relevant for your study

Problem: "Geographical and institutional barriers often limit meaningful interaction between those in different healthcare disciplines, as many educational programs are housed in separate departments or institutions[9]."
Type of solution: "Technology-Enabled IPE: Serious Games as a Solution"
Particular patient population: "undergraduate healthcare students"
Goals of the intervention: "We explore how experiential learning, utilizing immersive virtual reality and web-based platforms differentially influence both concrete learning outcomes and essential experiential factors."

2a-ii) Scientific background, rationale: What is known about the (type of) system

Scientific background, rationale: What is known about the (type of) system that is the object of the study (be sure to discuss the use of similar systems for other conditions/diagnoses, if appropiate), motivation for the study, i.e. what are the reasons for and what is the context for this specific study, from which stakeholder viewpoint is the study performed, potential impact of findings [2]. Briefly justify the choice of the comparator.

	1	2	3	4	5	
subitem not at all important	○	○	○	○	◉	essential

Clear selection

Does your paper address subitem 2a-ii? *

Copy and paste relevant sections from the manuscript (include quotes in quotation marks "like this" to indicate direct quotes from your manuscript), or elaborate on this item by providing additional information not in the ms, or briefly explain why the item is not applicable/relevant for your study

Scientific background, rationale: "A systematic review revealed that the most commonly used teaching and learning methods in universities to implement IPE were problem-based learning, case-based learning, and team-based learning[13]. In addition, simulation-based learning, e-learning, and serious games have been recognized as effective methods for cultivating IPE competencies[13-16]."
Motivation for the study: "Despite these established benefits, no studies have directly compared how these delivery modalities (IVR vs. web-based learning) influences IPE outcomes."

2b) In INTRODUCTION: Specific objectives or hypotheses

Does your paper address CONSORT subitem 2b? *

Copy and paste relevant sections from the manuscript (include quotes in quotation marks "like this" to indicate direct quotes from your manuscript), or elaborate on this item by providing additional information not in the ms, or briefly explain why the item is not applicable/relevant for your study

"Objectives
This study compared IVR versus web-based serious games in developing healthcare students' (1) interprofessional competencies (teamwork readiness, knowledge acquisition) and (2) learning experiences (motivation, community perception), with the aim of identifying the optimal modality for IPE implementation. The investigation specifically assessed how each approach differentially impacts competency development, intrinsic engagement (interest, perceived competence), and community formation (membership, support, connection), providing evidence for technology-enhanced IPE strategies."

METHODS

3a) Description of trial design (such as parallel, factorial) including allocation ratio

Does your paper address CONSORT subitem 3a? *

Copy and paste relevant sections from the manuscript (include quotes in quotation marks "like this" to indicate direct quotes from your manuscript), or elaborate on this item by providing additional information not in the ms, or briefly explain why the item is not applicable/relevant for your study

"Study Design
This study employed a multiple-methods design, which incorporated pre-/post-learning approaches to measure competency gains, knowledge-checking games to evaluate the application of skills, and focus group interviews to capture qualitative experiential differences between the two delivery modalities. "

3b) Important changes to methods after trial commencement (such as eligibility criteria), with reasons

Does your paper address CONSORT subitem 3b? *

Copy and paste relevant sections from the manuscript (include quotes in quotation marks "like this" to indicate direct quotes from your manuscript), or elaborate on this item by providing additional information not in the ms, or briefly explain why the item is not applicable/relevant for your study

"According to the trial protocol, a mixed 2 × 2 MANOVA was prespecified to explore the interactions between group differences and the within-subject factor. However, upon examining the data, the assumption of normality was violated. Consequently, as a deviation from the prespecified analysis, non-parametric tests were used."

3b-i) Bug fixes, Downtimes, Content Changes

Bug fixes, Downtimes, Content Changes: ehealth systems are often dynamic systems. A description of changes to methods therefore also includes important changes made on the intervention or comparator during the trial (e.g., major bug fixes or changes in the functionality or content) (5-iii) and other "unexpected events" that may have influenced study design such as staff changes, system failures/downtimes, etc. [2].

	1	2	3	4	5	
subitem not at all important	○	○	○	○	○	essential

Does your paper address subitem 3b-i?

Copy and paste relevant sections from the manuscript (include quotes in quotation marks "like this" to indicate direct quotes from your manuscript), or elaborate on this item by providing additional information not in the ms, or briefly explain why the item is not applicable/relevant for your study

Your answer

4a) Eligibility criteria for participants

Does your paper address CONSORT subitem 4a? *

Copy and paste relevant sections from the manuscript (include quotes in quotation marks "like this" to indicate direct quotes from your manuscript), or elaborate on this item by providing additional information not in the ms, or briefly explain why the item is not applicable/relevant for your study

"Convenience sampling was employed to recruit undergraduate healthcare students from the 2024/25 cohort. The participant pool consisted of 215 third-year nursing students enrolled in a gerontological nursing course and 68 third- and fourth-year physiotherapy (PT) students attending pre-clinical training workshops.
All eligible students were invited to complete surveys about their learning experiences as part of the course evaluation. However, only students who provided consent were included in the study analyses and reporting. Students were organized into 34 interprofessional groups, each comprising 7-8 nursing students and 1-2 PT students and randomly allocated to one of two learning modalities: IVR training (n=17 IPE groups) or a web-based serious gaming platform (n=17 IPE groups). Details of the randomization procedure are provided in the “Randomization” section."

4a-i) Computer / Internet literacy

Computer / Internet literacy is often an implicit “de facto” eligibility criterion - this should be explicitly clarified.

	1	2	3	4	5	
subitem not at all important	○	○	○	○	○	essential

Does your paper address subitem 4a-i?

Copy and paste relevant sections from the manuscript (include quotes in quotation marks "like this" to indicate direct quotes from your manuscript), or elaborate on this item by providing additional information not in the ms, or briefly explain why the item is not applicable/relevant for your study

Your answer

4a-ii) Open vs. closed, web-based vs. face-to-face assessments:

Open vs. closed, web-based vs. face-to-face assessments: Mention how participants were recruited (online vs. offline), e.g., from an open access website or from a clinic, and clarify if this was a purely web-based trial, or there were face-to-face components (as part of the intervention or for assessment), i.e., to what degree got the study team to know the participant. In online-only trials, clarify if participants were quasi-anonymous and whether having multiple identities was possible or whether technical or logistical measures (e.g., cookies, email confirmation, phone calls) were used to detect/prevent these.

	1	2	3	4	5	
subitem not at all important	○	○	○	○	◉	essential

Clear selection

Does your paper address subitem 4a-ii? *

Copy and paste relevant sections from the manuscript (include quotes in quotation marks "like this" to indicate direct quotes from your manuscript), or elaborate on this item by providing additional information not in the ms, or briefly explain why the item is not applicable/relevant for your study

"The IPE program was delivered through two modalities: A quick informal briefing and debriefing lasting 30 minutes was conducted with the participants. Seventeen IVR groups participated in a structured 2-hour face-to-face tutorial during the 7th week of a 13-week semester. The tutorial utilized five IVR setups to simulate clinical rehabilitation environments, where four to five IPE teams per tutorial rotated through game-based learning activities in a similar setup during each session. Meanwhile, the web-based groups accessed identical content through a web-based platform over the same week, at their convenience, enabling asynchronous collaboration without time or location constraints. "

4a-iii) Information giving during recruitment

Information given during recruitment. Specify how participants were briefed for recruitment and in the informed consent procedures (e.g., publish the informed consent documentation as appendix, see also item X26), as this information may have an effect on user self-selection, user expectation and may also bias results.

	1	2	3	4	5	
subitem not at all important	○	○	○	○	◉	essential

Clear selection

Does your paper address subitem 4a-iii?

Copy and paste relevant sections from the manuscript (include quotes in quotation marks "like this" to indicate direct quotes from your manuscript), or elaborate on this item by providing additional information not in the ms, or briefly explain why the item is not applicable/relevant for your study

Information given during recruitment: "Convenience sampling was employed to recruit undergraduate healthcare students from the 2024/25 cohort. The participant pool consisted of 215 third-year nursing students enrolled in a gerontological nursing course and 68 third- and fourth-year physiotherapy (PT) students attending pre-clinical training workshops."

Informed consent procedures: "All students were invited to complete surveys about their learning experiences, and participation in the focus groups was optional. As the surveys and the knowledge-check tests (MCQs) were self-administrated, blinding was not required."

4b) Settings and locations where the data were collected

Does your paper address CONSORT subitem 4b? *

Copy and paste relevant sections from the manuscript (include quotes in quotation marks "like this" to indicate direct quotes from your manuscript), or elaborate on this item by providing additional information not in the ms, or briefly explain why the item is not applicable/relevant for your study

Settings: "The IPE program was delivered through two modalities: A quick informal briefing and debriefing lasting 30 minutes was conducted with the participants."

Locations: "This study was conducted at a government-funded university that offers undergraduate nursing and physiotherapy programs."

4b-i) Report if outcomes were (self-)assessed through online questionnaires

Clearly report if outcomes were (self-)assessed through online questionnaires (as common in web-based trials) or otherwise.

	1	2	3	4	5	
subitem not at all important	○	○	○	○	◉	essential

Clear selection

Does your paper address subitem 4b-i? *

Copy and paste relevant sections from the manuscript (include quotes in quotation marks "like this" to indicate direct quotes from your manuscript), or elaborate on this item by providing additional information not in the ms, or briefly explain why the item is not applicable/relevant for your study

"All students were invited to complete surveys about their learning experiences, and participation in the focus groups was optional. As the surveys and the knowledge-check tests (MCQs) were self-administrated, blinding was not required."

4b-ii) Report how institutional affiliations are displayed

Report how institutional affiliations are displayed to potential participants [on ehealth media], as affiliations with prestigious hospitals or universities may affect volunteer rates, use, and reactions with regards to an intervention.(Not a required item – describe only if this may bias results)

	1	2	3	4	5	
subitem not at all important	○	○	○	○	○	essential

Does your paper address subitem 4b-ii?

Copy and paste relevant sections from the manuscript (include quotes in quotation marks "like this" to indicate direct quotes from your manuscript), or elaborate on this item by providing additional information not in the ms, or briefly explain why the item is not applicable/relevant for your study

Your answer

5) The interventions for each group with sufficient details to allow replication, including how and when they were actually administered

5-i) Mention names, credential, affiliations of the developers, sponsors, and owners

Mention names, credential, affiliations of the developers, sponsors, and owners [6] (if authors/evaluators are owners or developer of the software, this needs to be declared in a “Conflict of interest” section or mentioned elsewhere in the manuscript).

	1	2	3	4	5	
subitem not at all important	○	○	○	○	○	essential

Does your paper address subitem 5-i?

Copy and paste relevant sections from the manuscript (include quotes in quotation marks "like this" to indicate direct quotes from your manuscript), or elaborate on this item by providing additional information not in the ms, or briefly explain why the item is not applicable/relevant for your study

Your answer

5-ii) Describe the history/development process

Describe the history/development process of the application and previous formative evaluations (e.g., focus groups, usability testing), as these will have an impact on adoption/use rates and help with interpreting results.

	1	2	3	4	5	
subitem not at all important	○	○	○	○	○	essential

Does your paper address subitem 5-ii?

Copy and paste relevant sections from the manuscript (include quotes in quotation marks "like this" to indicate direct quotes from your manuscript), or elaborate on this item by providing additional information not in the ms, or briefly explain why the item is not applicable/relevant for your study

Your answer

5-iii) Revisions and updating

Revisions and updating. Clearly mention the date and/or version number of the application/intervention (and comparator, if applicable) evaluated, or describe whether the intervention underwent major changes during the evaluation process, or whether the development and/or content was “frozen” during the trial. Describe dynamic components such as news feeds or changing content which may have an impact on the replicability of the intervention (for unexpected events see item 3b).

	1	2	3	4	5	
subitem not at all important	○	○	○	○	○	essential

Does your paper address subitem 5-iii?

Copy and paste relevant sections from the manuscript (include quotes in quotation marks "like this" to indicate direct quotes from your manuscript), or elaborate on this item by providing additional information not in the ms, or briefly explain why the item is not applicable/relevant for your study

Your answer

5-iv) Quality assurance methods

Provide information on quality assurance methods to ensure accuracy and quality of information provided [1], if applicable.

	1	2	3	4	5	
subitem not at all important	○	○	○	○	○	essential

Does your paper address subitem 5-iv?

Copy and paste relevant sections from the manuscript (include quotes in quotation marks "like this" to indicate direct quotes from your manuscript), or elaborate on this item by providing additional information not in the ms, or briefly explain why the item is not applicable/relevant for your study

Your answer

5-v) Ensure replicability by publishing the source code, and/or providing screenshots/screen-capture video, and/or providing flowcharts of the algorithms used

Ensure replicability by publishing the source code, and/or providing screenshots/screen-capture video, and/or providing flowcharts of the algorithms used. Replicability (i.e., other researchers should in principle be able to replicate the study) is a hallmark of scientific reporting.

	1	2	3	4	5	
subitem not at all important	○	○	○	○	○	essential

Does your paper address subitem 5-v?

Copy and paste relevant sections from the manuscript (include quotes in quotation marks "like this" to indicate direct quotes from your manuscript), or elaborate on this item by providing additional information not in the ms, or briefly explain why the item is not applicable/relevant for your study

Your answer

5-vi) Digital preservation

Digital preservation: Provide the URL of the application, but as the intervention is likely to change or disappear over the course of the years; also make sure the intervention is archived (Internet Archive, webcitation.org, and/or publishing the source code or screenshots/videos alongside the article). As pages behind login screens cannot be archived, consider creating demo pages which are accessible without login.

	1	2	3	4	5	
subitem not at all important	○	○	○	○	○	essential

Does your paper address subitem 5-vi?

Copy and paste relevant sections from the manuscript (include quotes in quotation marks "like this" to indicate direct quotes from your manuscript), or elaborate on this item by providing additional information not in the ms, or briefly explain why the item is not applicable/relevant for your study

Your answer

5-vii) Access

Access: Describe how participants accessed the application, in what setting/context, if they had to pay (or were paid) or not, whether they had to be a member of specific group. If known, describe how participants obtained “access to the platform and Internet” [1]. To ensure access for editors/reviewers/readers, consider to provide a “backdoor” login account or demo mode for reviewers/readers to explore the application (also important for archiving purposes, see vi).

	1	2	3	4	5	
subitem not at all important	○	○	○	○	○	essential

Does your paper address subitem 5-vii? *

Copy and paste relevant sections from the manuscript (include quotes in quotation marks "like this" to indicate direct quotes from your manuscript), or elaborate on this item by providing additional information not in the ms, or briefly explain why the item is not applicable/relevant for your study

Accessed the application: "While participation in the IPE tutorials was mandatory for nursing and PT students, involvement in the research study was strictly voluntary. "
Access to the platform and Internet: "The tutorial utilized five IVR setups to simulate clinical rehabilitation environments, where four to five IPE teams per tutorial rotated through game-based learning activities in a similar setup during each session. "
Since the two serious games in the study are still in development, the data and access of the 2 platforms are not available nor would it be appropriate to share it at the current stage.

5-viii) Mode of delivery, features/functionalities/components of the intervention and comparator, and the theoretical framework

Describe mode of delivery, features/functionalities/components of the intervention and comparator, and the theoretical framework [6] used to design them (instructional strategy [1], behaviour change techniques, persuasive features, etc., see e.g., [7, 8] for terminology). This includes an in-depth description of the content (including where it is coming from and who developed it) [1]," whether [and how] it is tailored to individual circumstances and allows users to track their progress and receive feedback" [6]. This also includes a description of communication delivery channels and – if computer-mediated communication is a component – whether communication was synchronous or asynchronous [6]. It also includes information on presentation strategies [1], including page design principles, average amount of text on pages, presence of hyperlinks to other resources, etc. [1].

	1	2	3	4	5	
subitem not at all important	○	○	○	○	◉	essential

Clear selection

Does your paper address subitem 5-viii? *

Copy and paste relevant sections from the manuscript (include quotes in quotation marks "like this" to indicate direct quotes from your manuscript), or elaborate on this item by providing additional information not in the ms, or briefly explain why the item is not applicable/relevant for your study

"The IPE program was delivered through two modalities: A quick informal briefing and debriefing lasting 30 minutes was conducted with the participants. Seventeen IVR groups participated in a structured 2-hour face-to-face tutorial during the 7th week of a 13-week semester. The tutorial utilized five IVR setups to simulate clinical rehabilitation environments, where four to five IPE teams per tutorial rotated through game-based learning activities in a similar setup during each session. Meanwhile, the web-based groups accessed identical content through a web-based platform over the same week, at their convenience, enabling asynchronous collaboration without time or location constraints. "

5-ix) Describe use parameters

Describe use parameters (e.g., intended "doses" and optimal timing for use). Clarify what instructions or recommendations were given to the user, e.g., regarding timing, frequency, heaviness of use, if any, or was the intervention used ad libitum.

	1	2	3	4	5	
subitem not at all important	○	○	○	○	○	essential

Does your paper address subitem 5-ix?

Copy and paste relevant sections from the manuscript (include quotes in quotation marks "like this" to indicate direct quotes from your manuscript), or elaborate on this item by providing additional information not in the ms, or briefly explain why the item is not applicable/relevant for your study

Your answer

5-x) Clarify the level of human involvement

Clarify the level of human involvement (care providers or health professionals, also technical assistance) in the e-intervention or as co-intervention (detail number and expertise of professionals involved, if any, as well as “type of assistance offered, the timing and frequency of the support, how it is initiated, and the medium by which the assistance is delivered”. It may be necessary to distinguish between the level of human involvement required for the trial, and the level of human involvement required for a routine application outside of a RCT setting (discuss under item 21 – generalizability).

	1	2	3	4	5	
subitem not at all important	○	○	○	○	○	essential

Does your paper address subitem 5-x?

Copy and paste relevant sections from the manuscript (include quotes in quotation marks "like this" to indicate direct quotes from your manuscript), or elaborate on this item by providing additional information not in the ms, or briefly explain why the item is not applicable/relevant for your study

Your answer

5-xi) Report any prompts/reminders used

Report any prompts/reminders used: Clarify if there were prompts (letters, emails, phone calls, SMS) to use the application, what triggered them, frequency etc. It may be necessary to distinguish between the level of prompts/reminders required for the trial, and the level of prompts/reminders for a routine application outside of a RCT setting (discuss under item 21 – generalizability).

	1	2	3	4	5	
subitem not at all important	○	○	○	○	○	essential

Does your paper address subitem 5-xi? *

Copy and paste relevant sections from the manuscript (include quotes in quotation marks "like this" to indicate direct quotes from your manuscript), or elaborate on this item by providing additional information not in the ms, or briefly explain why the item is not applicable/relevant for your study

As the intervention is embedded in the course, no prompts/reminders is sent as student should find all information from the learning platforms but they could ask the lectures during class or tutorial session if any assist is needed. However, this does not related to the study directly, hence, we did not mentioned in the main text.

5-xii) Describe any co-interventions (incl. training/support)

Describe any co-interventions (incl. training/support): Clearly state any interventions that are provided in addition to the targeted eHealth intervention, as ehealth intervention may not be designed as stand-alone intervention. This includes training sessions and support [1]. It may be necessary to distinguish between the level of training required for the trial, and the level of training for a routine application outside of a RCT setting (discuss under item 21 – generalizability.

	1	2	3	4	5	
subitem not at all important	○	○	○	◉	○	essential

Clear selection

Does your paper address subitem 5-xii? *

Copy and paste relevant sections from the manuscript (include quotes in quotation marks "like this" to indicate direct quotes from your manuscript), or elaborate on this item by providing additional information not in the ms, or briefly explain why the item is not applicable/relevant for your study

There was a "pre-clinical training workshops at a local university" in the beginning of the semester.

6a) Completely defined pre-specified primary and secondary outcome measures, including how and when they were assessed

Does your paper address CONSORT subitem 6a? *

Copy and paste relevant sections from the manuscript (include quotes in quotation marks "like this" to indicate direct quotes from your manuscript), or elaborate on this item by providing additional information not in the ms, or briefly explain why the item is not applicable/relevant for your study

"Outcome Measurement
Participants were assessed at two strategic timepoints to capture both modality-specific and comparative learning outcomes. Following completion of their first assigned learning approach (either IVR or a web-based platform) but prior to experiencing the alternative modality, all participants completed self-administered surveys where the knowledge checking activities were embedded in the IPE learning games."

6a-i) Online questionnaires: describe if they were validated for online use and apply CHERRIES items to describe how the questionnaires were designed/deployed

If outcomes were obtained through online questionnaires, describe if they were validated for online use and apply CHERRIES items to describe how the questionnaires were designed/deployed [9].

	1	2	3	4	5	
subitem not at all important	○	○	○	○	◉	essential

Clear selection

Does your paper address subitem 6a-i?

Copy and paste relevant sections from manuscript text

"Participants were assessed at two strategic timepoints to capture both modality-specific and comparative learning outcomes. Following completion of their first assigned learning approach (either IVR or a web-based platform) but prior to experiencing the alternative modality, all participants completed self-administered surveys where the knowledge checking activities were embedded in the IPE learning games. "

6a-ii) Describe whether and how “use” (including intensity of use/dosage) was defined/measured/monitored

Describe whether and how “use” (including intensity of use/dosage) was defined/measured/monitored (logins, logfile analysis, etc.). Use/adoption metrics are important process outcomes that should be reported in any ehealth trial.

	1	2	3	4	5	
subitem not at all important	○	○	○	○	○	essential

Does your paper address subitem 6a-ii?

Copy and paste relevant sections from manuscript text

Your answer

6a-iii) Describe whether, how, and when qualitative feedback from participants was obtained

Describe whether, how, and when qualitative feedback from participants was obtained (e.g., through emails, feedback forms, interviews, focus groups).

	1	2	3	4	5	
subitem not at all important	○	○	○	○	◉	essential

Clear selection

Does your paper address subitem 6a-iii?

Copy and paste relevant sections from manuscript text

"Participation in the focus groups was optional. After completion of the IPE learning activities, four face-to-face focus group discussions were conducted, with each session lasting an average of approximately 60 minutes. Representatives from 34 groups were invited to participate in the focus group, which involved 34 students (22 nursing and 12 PT students). "

6b) Any changes to trial outcomes after the trial commenced, with reasons

Does your paper address CONSORT subitem 6b? *

Copy and paste relevant sections from the manuscript (include quotes in quotation marks "like this" to indicate direct quotes from your manuscript), or elaborate on this item by providing additional information not in the ms, or briefly explain why the item is not applicable/relevant for your study

"According to the trial protocol, a mixed 2 × 2 MANOVA was prespecified to explore the interactions between group differences and the within-subject factor. However, upon examining the data, the assumption of normality was violated. Consequently, as a deviation from the prespecified analysis, non-parametric tests were used. "

7a) How sample size was determined

NPT: When applicable, details of whether and how the clustering by care provides or centers was addressed

7a-i) Describe whether and how expected attrition was taken into account when calculating the sample size

Describe whether and how expected attrition was taken into account when calculating the sample size.

	1	2	3	4	5	
subitem not at all important	○	○	○	○	◉	essential

Clear selection

Does your paper address subitem 7a-i?

Copy and paste relevant sections from manuscript title (include quotes in quotation marks "like this" to indicate direct quotes from your manuscript), or elaborate on this item by providing additional information not in the ms, or briefly explain why the item is not applicable/relevant for your study

"A power analysis determined the sample size. Previous studies of similar educational interventions reported medium to large effect sizes in learning outcomes from pre- to post-intervention[35-37]. Adopting a conservative approach, we based our power analysis on detecting a medium effect using a 2 x 2 mixed multivariate analysis of variance (MANOVA). The results indicated that a sample size of 128 is sufficient to detect a medium two-tailed effect at a significance level of p<0.05 with 80% power. We anticipate a sample size of around 150-160 to account for potential missing data. Based on the nurse-physiotherapist ratio, this would translate to approximately 112-128 student nurses and 32-38 student physiotherapists."

7b) When applicable, explanation of any interim analyses and stopping guidelines

Does your paper address CONSORT subitem 7b? *

Copy and paste relevant sections from the manuscript (include quotes in quotation marks "like this" to indicate direct quotes from your manuscript), or elaborate on this item by providing additional information not in the ms, or briefly explain why the item is not applicable/relevant for your study

This section is not applicable. There is only a stopping guideline for harms: "The potential harms associated with the intervention were minimal. One possible adverse effect was transient dizziness or nausea (i.e., virtual reality sickness) associated with prolonged IVR use. Both students and lecturers were informed of this risk. Students were instructed to stop the activity immediately and report any physiological discomfort to the lecturers so that appropriate support could be provided. If symptoms occurred, students were allowed to take a break or withdraw from the session as needed."

8a) Method used to generate the random allocation sequence

NPT: When applicable, how care providers were allocated to each trial group

Does your paper address CONSORT subitem 8a? *

Copy and paste relevant sections from the manuscript (include quotes in quotation marks "like this" to indicate direct quotes from your manuscript), or elaborate on this item by providing additional information not in the ms, or briefly explain why the item is not applicable/relevant for your study

"Randomization

Students were organized into 34 interprofessional groups, each comprising 7-8 nursing students and 1-2 PT students. A randomization sequence was conducted at the group level for administrative purposes. A computer-generated random sequence (1:1 allocation) was used to allocate the 34 IPE to either IVR training (n=17 IPE groups) or a web-based serious gaming platform (n=17 IPE groups). The allocation sequence was generated and implemented by the lecturers (JL and CW). "

8b) Type of randomisation; details of any restriction (such as blocking and block size)

Does your paper address CONSORT subitem 8b? *

Copy and paste relevant sections from the manuscript (include quotes in quotation marks "like this" to indicate direct quotes from your manuscript), or elaborate on this item by providing additional information not in the ms, or briefly explain why the item is not applicable/relevant for your study

"Randomization
Students were organized into 34 interprofessional groups, each comprising 7-8 nursing students and 1-2 PT students. A randomization sequence was conducted at the group level for administrative purposes. A computer-generated random sequence (1:1 allocation) was used to allocate the 34 IPE to either IVR training (n=17 IPE groups) or a web-based serious gaming platform (n=17 IPE groups). The allocation sequence was generated and implemented by the lecturers (JL and CW). Due to the nature of the study design, students were aware of the group to which they had been assigned. Following a crossover design, the groups completed all of the required assignments on their initial learning approach for one week before switching to the alternative modality. All students were invited to complete surveys about their learning experiences, and participation in the focus groups was optional. As the surveys and the knowledge-check tests (MCQs) were self-administrated, blinding was not required. "

9) Mechanism used to implement the random allocation sequence (such as sequentially numbered containers), describing any steps taken to conceal the sequence until interventions were assigned

Does your paper address CONSORT subitem 9? *

Copy and paste relevant sections from the manuscript (include quotes in quotation marks "like this" to indicate direct quotes from your manuscript), or elaborate on this item by providing additional information not in the ms, or briefly explain why the item is not applicable/relevant for your study

"Randomization
A randomization sequence was conducted at the group level for administrative purposes. A computer-generated random sequence (1:1 allocation) was used to allocate the 34 IPE to either IVR training (n=17 IPE groups) or a web-based serious gaming platform (n=17 IPE groups). The allocation sequence was generated and implemented by the lecturers (JL and CW). "

10) Who generated the random allocation sequence, who enrolled participants, and who assigned participants to interventions

Does your paper address CONSORT subitem 10? *

Copy and paste relevant sections from the manuscript (include quotes in quotation marks "like this" to indicate direct quotes from your manuscript), or elaborate on this item by providing additional information not in the ms, or briefly explain why the item is not applicable/relevant for your study

"The allocation sequence was generated and implemented by the lecturers (JL and CW). "

11a) If done, who was blinded after assignment to interventions (for example, participants, care providers, those assessing outcomes) and how

NPT: Whether or not administering co-interventions were blinded to group assignment

11a-i) Specify who was blinded, and who wasn't

Specify who was blinded, and who wasn't. Usually, in web-based trials it is not possible to blind the participants [1, 3] (this should be clearly acknowledged), but it may be possible to blind outcome assessors, those doing data analysis or those administering co-interventions (if any).

	1	2	3	4	5	
subitem not at all important	○	○	○	○	◉	essential

Clear selection

Does your paper address subitem 11a-i? *

Copy and paste relevant sections from the manuscript (include quotes in quotation marks "like this" to indicate direct quotes from your manuscript), or elaborate on this item by providing additional information not in the ms, or briefly explain why the item is not applicable/relevant for your study

"As the surveys and the knowledge-check tests (MCQs) were self-administrated, blinding was not required."

11a-ii) Discuss e.g., whether participants knew which intervention was the "intervention of interest" and which one was the "comparator"

Informed consent procedures (4a-ii) can create biases and certain expectations - discuss e.g., whether participants knew which intervention was the "intervention of interest" and which one was the "comparator".

	1	2	3	4	5	
subitem not at all important	○	○	○	○	○	essential

Does your paper address subitem 11a-ii?

Copy and paste relevant sections from the manuscript (include quotes in quotation marks "like this" to indicate direct quotes from your manuscript), or elaborate on this item by providing additional information not in the ms, or briefly explain why the item is not applicable/relevant for your study

Your answer

11b) If relevant, description of the similarity of interventions

(this item is usually not relevant for ehealth trials as it refers to similarity of a placebo or sham intervention to a active medication/intervention)

Does your paper address CONSORT subitem 11b? *

Copy and paste relevant sections from the manuscript (include quotes in quotation marks "like this" to indicate direct quotes from your manuscript), or elaborate on this item by providing additional information not in the ms, or briefly explain why the item is not applicable/relevant for your study

"This identical clinical scenario was delivered through both modalities [IVR (Figure 1) and web-based platforms (Figure 2)], maintaining consistency in learning objectives across conditions. The web-based platforms had different UI panels for the PC and mobile displays, with a website URL that made both the PC and mobile platforms user-friendly. The two formats differed only in their technological delivery and gamification elements, with content that did not require hands-on practice (Table 1)."

12a) Statistical methods used to compare groups for primary and secondary outcomes

NPT: When applicable, details of whether and how the clustering by care providers or centers was addressed

Does your paper address CONSORT subitem 12a? *

Copy and paste relevant sections from the manuscript (include quotes in quotation marks "like this" to indicate direct quotes from your manuscript), or elaborate on this item by providing additional information not in the ms, or briefly explain why the item is not applicable/relevant for your study

"Quantitative data
IBM SPSS 29.0 software was used to analyze the quantitative data. With regard to descriptive statistics, mean and standard deviation were adopted for continuous data, and frequency and percentage for categorical variables. A Chi-squared test or Fisher’s Exact Test was used for categorical variables. According to the trial protocol, a mixed 2 × 2 MANOVA was prespecified to explore the interactions between group differences and the within-subject factor. However, upon examining the data, the assumption of normality was violated. Consequently, as a deviation from the prespecified analysis, non-parametric tests were used. The median and interquartile range were used to present outcome measures. Within-group differences were analyzed using the Wilcoxon Signed Rank Test, while the Mann-Whitney U Test was applied to analyze the between-group differences. To strengthen the interpretation of the results, effect sizes (r) and confidence intervals were adopted. The level of significance was set at .05. Samples with missing data were excluded from the analysis."

12a-i) Imputation techniques to deal with attrition / missing values

Imputation techniques to deal with attrition / missing values: Not all participants will use the intervention/comparator as intended and attrition is typically high in ehealth trials. Specify how participants who did not use the application or dropped out from the trial were treated in the statistical analysis (a complete case analysis is strongly discouraged, and simple imputation techniques such as LOCF may also be problematic [4]).

	1	2	3	4	5	
subitem not at all important	○	○	○	○	◉	essential

Clear selection

Does your paper address subitem 12a-i? *

Copy and paste relevant sections from the manuscript (include quotes in quotation marks "like this" to indicate direct quotes from your manuscript), or elaborate on this item by providing additional information not in the ms, or briefly explain why the item is not applicable/relevant for your study

"Only those who consented to join the study were included in the analysis and reporting of the results. "

12b) Methods for additional analyses, such as subgroup analyses and adjusted analyses

Does your paper address CONSORT subitem 12b? *

Copy and paste relevant sections from the manuscript (include quotes in quotation marks "like this" to indicate direct quotes from your manuscript), or elaborate on this item by providing additional information not in the ms, or briefly explain why the item is not applicable/relevant for your study

"Qualitative analysis
Qualitative data were collected through focus group interviews. The semi-structured interviews, which employed overarching questions, were transcribed verbatim and analyzed using thematic analysis (please see Appendix B for the interview questions). The process of analysis began with initial open coding, during which significant statements and phrases were identified for further thematic development. These codes were subsequently grouped with meaningful sub-themes. Lastly, the sub-themes were condensed to form overarching themes, leading to the identification of key themes. To ensure qualitative rigor, all interview transcripts were independently coded by two researchers (RW and JL). The initial codes were compared and discussed to ensure consistency, and any discrepancies that could not be resolved were reviewed by a third researcher (KC) to reach a consensus. The development of key themes followed a similar iterative process, involving comparison, discussion, and consensus among the two researchers (RW and JL) to ensure the credibility and trustworthiness of the findings."

X26) REB/IRB Approval and Ethical Considerations [recommended as subheading under "Methods"] (not a CONSORT item)

X26-i) Comment on ethics committee approval

	1	2	3	4	5	
subitem not at all important	○	○	○	○	◉	essential

Clear selection

Does your paper address subitem X26-i?

Copy and paste relevant sections from the manuscript (include quotes in quotation marks "like this" to indicate direct quotes from your manuscript), or elaborate on this item by providing additional information not in the ms, or briefly explain why the item is not applicable/relevant for your study

"Ethical Considerations
Ethical approval was obtained from the Human Subjects Ethics Review Committee of a university (HSEARS20240827002). While participation in the IPE tutorials was mandatory for nursing and PT students, involvement in the research study was strictly voluntary. Students were explicitly assured that their decision to participate would not affect their academic standing, course grades, or access to learning opportunities. No monetary or nonmonetary compensation, course credit, or other incentives were provided for participation in the research study. Written informed consent was obtained from all study participants, and stringent confidentiality measures were implemented, including de-identification of all data prior to analysis. Access to raw data was restricted to only three nursing faculty members (JL, PK, SSW), the PT lecturer (CW), and the project research assistant (RW) to protect participant privacy while enabling necessary educational assessments. This clear separation between required learning components and optional research participation was maintained throughout the study period, with all students receiving equal access to learning experiences regardless of their research participation status. In addition, no identification of individual participants in this study is possible in any images included in the paper or supplementary materials."

x26-ii) Outline informed consent procedures

Outline informed consent procedures e.g., if consent was obtained offline or online (how? Checkbox, etc.?), and what information was provided (see 4a-ii). See [6] for some items to be included in informed consent documents.

	1	2	3	4	5	
subitem not at all important	○	○	○	○	○	essential

Does your paper address subitem X26-ii?

Copy and paste relevant sections from the manuscript (include quotes in quotation marks "like this" to indicate direct quotes from your manuscript), or elaborate on this item by providing additional information not in the ms, or briefly explain why the item is not applicable/relevant for your study

Your answer

X26-iii) Safety and security procedures

Safety and security procedures, incl. privacy considerations, and any steps taken to reduce the likelihood or detection of harm (e.g., education and training, availability of a hotline)

	1	2	3	4	5	
subitem not at all important	○	○	○	○	○	essential

Does your paper address subitem X26-iii?

Copy and paste relevant sections from the manuscript (include quotes in quotation marks "like this" to indicate direct quotes from your manuscript), or elaborate on this item by providing additional information not in the ms, or briefly explain why the item is not applicable/relevant for your study

Your answer

RESULTS

13a) For each group, the numbers of participants who were randomly assigned, received intended treatment, and were analysed for the primary outcome

NPT: The number of care providers or centers performing the intervention in each group and the number of patients treated by each care provider in each center

Does your paper address CONSORT subitem 13a? *

Copy and paste relevant sections from the manuscript (include quotes in quotation marks "like this" to indicate direct quotes from your manuscript), or elaborate on this item by providing additional information not in the ms, or briefly explain why the item is not applicable/relevant for your study

"Baseline Data
The majority of the participants were female, aged between 19 and 21 years, and possessed experience with online gaming. Significant differences in age distribution (P=.001) and course enrollment (P<.001) were noted between the IVR and web-based groups (Table 2). There were no significant differences in terms of gender, profession, online gaming experience, or daily playtime. In addition, no significant between-group differences were identified for the outcome measures at baseline (Multimedia Appendix 4)."

13b) For each group, losses and exclusions after randomisation, together with reasons

Does your paper address CONSORT subitem 13b? (NOTE: Preferably, this is shown in a CONSORT flow diagram) *

Copy and paste relevant sections from the manuscript (include quotes in quotation marks "like this" to indicate direct quotes from your manuscript), or elaborate on this item by providing additional information not in the ms, or briefly explain why the item is not applicable/relevant for your study

As there only the students who signed the consent and completed the pre- and post-assessment was included in the study, there was no losses and exclusions after randomization. "A total of 283 students were invited to participate. Of these, 271 students (125 in the IVR group and 146 in the web-based group) consented to join the study, yielding a response rate of 95.76%. Participant flow through the trial is shown in Figure 3. The final sample consisted of 203 nursing students and 68 PT students. "

13b-i) Attrition diagram

Strongly recommended: An attrition diagram (e.g., proportion of participants still logging in or using the intervention/comparator in each group plotted over time, similar to a survival curve) or other figures or tables demonstrating usage/dose/engagement.

	1	2	3	4	5	
subitem not at all important	○	○	○	○	○	essential

Does your paper address subitem 13b-i?

Copy and paste relevant sections from the manuscript or cite the figure number if applicable (include quotes in quotation marks "like this" to indicate direct quotes from your manuscript), or elaborate on this item by providing additional information not in the ms, or briefly explain why the item is not applicable/relevant for your study

Your answer

14a) Dates defining the periods of recruitment and follow-up

Does your paper address CONSORT subitem 14a? *

Copy and paste relevant sections from the manuscript (include quotes in quotation marks "like this" to indicate direct quotes from your manuscript), or elaborate on this item by providing additional information not in the ms, or briefly explain why the item is not applicable/relevant for your study

Periods of recruitment: "Convenience sampling was employed to recruit undergraduate healthcare students from the 2024/25 cohort"
Follow-up: The IPE learning activities were strategically implemented during Weeks 7–8 of the 13-week semester, to ensure that the students would have acquired foundational knowledge before engaging in collaborative competency development."

14a-i) Indicate if critical “secular events” fell into the study period

Indicate if critical “secular events” fell into the study period, e.g., significant changes in Internet resources available or “changes in computer hardware or Internet delivery resources”

	1	2	3	4	5	
subitem not at all important	○	○	○	○	○	essential

Does your paper address subitem 14a-i?

Copy and paste relevant sections from the manuscript (include quotes in quotation marks "like this" to indicate direct quotes from your manuscript), or elaborate on this item by providing additional information not in the ms, or briefly explain why the item is not applicable/relevant for your study

Your answer

14b) Why the trial ended or was stopped (early)

Does your paper address CONSORT subitem 14b? *

Copy and paste relevant sections from the manuscript (include quotes in quotation marks "like this" to indicate direct quotes from your manuscript), or elaborate on this item by providing additional information not in the ms, or briefly explain why the item is not applicable/relevant for your study

Not applicable. The study is completed according to protocol.

15) A table showing baseline demographic and clinical characteristics for each group

NPT: When applicable, a description of care providers (case volume, qualification, expertise, etc.) and centers (volume) in each group

Does your paper address CONSORT subitem 15? *

Copy and paste relevant sections from the manuscript (include quotes in quotation marks "like this" to indicate direct quotes from your manuscript), or elaborate on this item by providing additional information not in the ms, or briefly explain why the item is not applicable/relevant for your study

"Table 2. Demographic data of the participants in the IVR and web-based groups in a randomized control trial of an IPE training program among undergraduate healthcare students at a local university"

15-i) Report demographics associated with digital divide issues

In ehealth trials it is particularly important to report demographics associated with digital divide issues, such as age, education, gender, social-economic status, computer/Internet/ehealth literacy of the participants, if known.

	1	2	3	4	5	
subitem not at all important	○	○	○	○	◉	essential

Clear selection

Does your paper address subitem 15-i? *

Copy and paste relevant sections from the manuscript (include quotes in quotation marks "like this" to indicate direct quotes from your manuscript), or elaborate on this item by providing additional information not in the ms, or briefly explain why the item is not applicable/relevant for your study

"Baseline Data
The majority of the participants were female, aged between 19 and 21 years, and possessed experience with online gaming. Significant differences in age distribution (P=.001) and course enrollment (P<.001) were noted between the IVR and web-based groups (Table 2). There were no significant differences in terms of gender, profession, online gaming experience, or daily playtime. In addition, no significant between-group differences were identified for the outcome measures at baseline (Multimedia Appendix 4)."

16) For each group, number of participants (denominator) included in each analysis and whether the analysis was by original assigned groups

16-i) Report multiple “denominators” and provide definitions

Report multiple “denominators” and provide definitions: Report N’s (and effect sizes) “across a range of study participation [and use] thresholds” [1], e.g., N exposed, N consented, N used more than x times, N used more than y weeks, N participants “used” the intervention/comparator at specific pre-defined time points of interest (in absolute and relative numbers per group). Always clearly define “use” of the intervention.

	1	2	3	4	5	
subitem not at all important	○	○	○	○	◉	essential

Clear selection

Does your paper address subitem 16-i? *

Copy and paste relevant sections from the manuscript (include quotes in quotation marks "like this" to indicate direct quotes from your manuscript), or elaborate on this item by providing additional information not in the ms, or briefly explain why the item is not applicable/relevant for your study

"Numbers analyzed outcomes and estimation for quantitative outcomes
Primary Outcomes
Within-group comparisons of the RIPLS, BSCS, IMI, and MCQ scores of the IVR group
No significant differences were noted in the RIPLS scores. Participants demonstrated significant improvements in the BSCS, 1 out of 3 subscales of IMI, and in the knowledge-check MCQs (Table 3a). A significant increase was seen in total BSCS scores (P=.001, r=-0.29, 95% CI 0.06 to 0.31), and in the dimensions of "Influence" (P<.001, r=-0.32, 95% CI 0.00 to 0.50), and "Emotional Connection" (P<.001, r=-0.33, 95% CI 0.25 to 0.50) (Supplementary Table 3). A significant decrease was noted in the score for the "pressure/tension" subscale of IMI (P<.001, r=-0.45, 95% CI -0.05 to -0.25). The IVR group (P<.001, r=-0.64, 95% CI 2.00 to 3.00) exhibited a significant improvement in their knowledge-check MCQs scores.

Within-group comparisons of the RIPLS, BSCS, IMI, and MCQ scores of the web-based group
No significant differences were noted in the RIPLS scores. Participants demonstrated significant improvements in the BSCS, all three subscales of IMI, and the knowledge-check MCQs scores (Table 3b). A significant increase was seen in the total BSCS scores (P=.001, r=-0.27, 95% CI 0.07 to 0.31) and in those for the dimensions of "Influence" (P<.001, r=-0.30, 95% CI 0.00 to 0.50), "Emotional Connection" (P=.004, r=-0.24, 95% CI 0.00 to 0.50), and "Needs fulfilment" (P=.03, r=-0.18, 95% CI 0.00 to 0.50). Significant increases in scores were observed in the IMI subscales of "interest/enjoyment" (P=.043, r=-0.17, 95% CI 0.00 to 0.30), and "perceived competence" (P=.022, r=-0.19, 95% CI 0.00 to 0.50), while significant decreases were revealed in the scores for the subscale of “pressure/tension" (P<.001, r=-0.46, 95% CI -0.75 to -0.50). Significant improvement was noted in the knowledge-check MCQs scores (P<.001, r=-0.80, 95% CI 4.50 to 6.00) (Table 3b).

Comparisons of the RIPLS, BSCS, IMI, and MCQ scores between the IVR and web-based groups
The web-based group demonstrated superior performance in knowledge-check MCQs compared with the IVR group (IVR median score:15.00, web-based media score:18.00, 95% CI -3.00 to -2.00, P<.001, r=-0.34). No significant differences were observed between the IVR and web-based groups in their total scores on the RIPLS, BSCS, or IMI outcomes at the post-assessment stage (see Table 4)."

16-ii) Primary analysis should be intent-to-treat

Primary analysis should be intent-to-treat, secondary analyses could include comparing only “users”, with the appropriate caveats that this is no longer a randomized sample (see 18-i).

	1	2	3	4	5	
subitem not at all important	○	○	○	○	○	essential

Does your paper address subitem 16-ii?

Copy and paste relevant sections from the manuscript (include quotes in quotation marks "like this" to indicate direct quotes from your manuscript), or elaborate on this item by providing additional information not in the ms, or briefly explain why the item is not applicable/relevant for your study

Your answer

17a) For each primary and secondary outcome, results for each group, and the estimated effect size and its precision (such as 95% confidence interval)

Does your paper address CONSORT subitem 17a? *

Copy and paste relevant sections from the manuscript (include quotes in quotation marks "like this" to indicate direct quotes from your manuscript), or elaborate on this item by providing additional information not in the ms, or briefly explain why the item is not applicable/relevant for your study

All tables reported median (IQR), 95% Confidence Interval, Effect Size (r), z score and p-value.
"Table 3a. Within-group pre-post comparisons of primary outcome measures in the IVR group in a randomized controlled trial of an IPE training program among undergraduate healthcare students at a local university"
"Table 3b. Within-group pre-post comparisons of primary outcome measures in the web-based group in a randomized controlled trial of an IPE training program among undergraduate healthcare students at a local university"
"Table 4. Between-group comparisons of primary outcome measures after the intervention in a randomized controlled trial of an IPE training program among undergraduate healthcare students at a local university"

17a-i) Presentation of process outcomes such as metrics of use and intensity of use

In addition to primary/secondary (clinical) outcomes, the presentation of process outcomes such as metrics of use and intensity of use (dose, exposure) and their operational definitions is critical. This does not only refer to metrics of attrition (13-b) (often a binary variable), but also to more continuous exposure metrics such as “average session length”. These must be accompanied by a technical description how a metric like a “session” is defined (e.g., timeout after idle time) [1] (report under item 6a).

	1	2	3	4	5	
subitem not at all important	○	○	○	○	○	essential

Does your paper address subitem 17a-i?

Copy and paste relevant sections from the manuscript (include quotes in quotation marks "like this" to indicate direct quotes from your manuscript), or elaborate on this item by providing additional information not in the ms, or briefly explain why the item is not applicable/relevant for your study

Your answer

17b) For binary outcomes, presentation of both absolute and relative effect sizes is recommended

Does your paper address CONSORT subitem 17b? *

Copy and paste relevant sections from the manuscript (include quotes in quotation marks "like this" to indicate direct quotes from your manuscript), or elaborate on this item by providing additional information not in the ms, or briefly explain why the item is not applicable/relevant for your study

There were no binary outcomes in the primary outcomes which all outcome measures were continuous variables.

18) Results of any other analyses performed, including subgroup analyses and adjusted analyses, distinguishing pre-specified from exploratory

Does your paper address CONSORT subitem 18? *

Copy and paste relevant sections from the manuscript (include quotes in quotation marks "like this" to indicate direct quotes from your manuscript), or elaborate on this item by providing additional information not in the ms, or briefly explain why the item is not applicable/relevant for your study

"Focus group participants
Referring to Table 5, a total 34 participants were engaged in the focus groups, most of whom were female, aged 19-21 years old, studying nursing, had experience with online games, and had prior experience with IPE. The average rating on overall experience was 5.78 (SD 1.97) out of a 10-point score, while that on IVR and web-based learning was 6.03 (SD 2.24) and 5.47 (SD 2.13), respectively. "
"Four focus group interviews were conducted, involving a total of 34 participants engaged with both the IVR and web-based groups. "

18-i) Subgroup analysis of comparing only users

A subgroup analysis of comparing only users is not uncommon in ehealth trials, but if done, it must be stressed that this is a self-selected sample and no longer an unbiased sample from a randomized trial (see 16-iii).

	1	2	3	4	5	
subitem not at all important	○	○	○	○	○	essential

Does your paper address subitem 18-i?

Copy and paste relevant sections from the manuscript (include quotes in quotation marks "like this" to indicate direct quotes from your manuscript), or elaborate on this item by providing additional information not in the ms, or briefly explain why the item is not applicable/relevant for your study

Your answer

19) All important harms or unintended effects in each group

(for specific guidance see CONSORT for harms)

Does your paper address CONSORT subitem 19? *

Copy and paste relevant sections from the manuscript (include quotes in quotation marks "like this" to indicate direct quotes from your manuscript), or elaborate on this item by providing additional information not in the ms, or briefly explain why the item is not applicable/relevant for your study

"Harms
No adverse events were reported by students in the study."

19-i) Include privacy breaches, technical problems

Include privacy breaches, technical problems. This does not only include physical "harm" to participants, but also incidents such as perceived or real privacy breaches [1], technical problems, and other unexpected/unintended incidents. "Unintended effects" also includes unintended positive effects [2].

	1	2	3	4	5	
subitem not at all important	○	○	○	○	○	essential

Does your paper address subitem 19-i?

Copy and paste relevant sections from the manuscript (include quotes in quotation marks "like this" to indicate direct quotes from your manuscript), or elaborate on this item by providing additional information not in the ms, or briefly explain why the item is not applicable/relevant for your study

Your answer

19-ii) Include qualitative feedback from participants or observations from staff/researchers

Include qualitative feedback from participants or observations from staff/researchers, if available, on strengths and shortcomings of the application, especially if they point to unintended/unexpected effects or uses. This includes (if available) reasons for why people did or did not use the application as intended by the developers.

	1	2	3	4	5	
subitem not at all important	○	○	○	○	○	essential

Does your paper address subitem 19-ii?

Copy and paste relevant sections from the manuscript (include quotes in quotation marks "like this" to indicate direct quotes from your manuscript), or elaborate on this item by providing additional information not in the ms, or briefly explain why the item is not applicable/relevant for your study

Your answer

DISCUSSION

22) Interpretation consistent with results, balancing benefits and harms, and considering other relevant evidence

NPT: In addition, take into account the choice of the comparator, lack of or partial blinding, and unequal expertise of care providers or centers in each group

22-i) Restate study questions and summarize the answers suggested by the data, starting with primary outcomes and process outcomes (use)

Restate study questions and summarize the answers suggested by the data, starting with primary outcomes and process outcomes (use).

	1	2	3	4	5	
subitem not at all important	○	○	○	○	◉	essential

Clear selection

Does your paper address subitem 22-i? *

Copy and paste relevant sections from the manuscript (include quotes in quotation marks "like this" to indicate direct quotes from your manuscript), or elaborate on this item by providing additional information not in the ms, or briefly explain why the item is not applicable/relevant for your study

Restate study questions: "To our knowledge, this is the first study to directly compare the efficacy of IVR and web-based serious games in fostering interprofessional competencies and learning experiences among healthcare students.
Summarize the answers suggested by the data, starting with primary outcomes and process outcomes: "Both approaches yielded notable improvements in community building, motivation, and knowledge acquisition, although no significant changes were observed in readiness for interprofessional teamwork. The web-based approach led to comparatively stronger gains in knowledge clarity and motivation, possibly due to its flexible and diversified learning formats that cater to different learning preferences so as to support knowledge retention. Overall, the findings suggest that both modalities hold practical value in advancing technology-enhanced interprofessional education, with distinct strengths depending on learning context and objectives."

22-ii) Highlight unanswered new questions, suggest future research

Highlight unanswered new questions, suggest future research.

	1	2	3	4	5	
subitem not at all important	○	○	○	○	○	essential

Does your paper address subitem 22-ii?

Copy and paste relevant sections from the manuscript (include quotes in quotation marks "like this" to indicate direct quotes from your manuscript), or elaborate on this item by providing additional information not in the ms, or briefly explain why the item is not applicable/relevant for your study

Your answer

20) Trial limitations, addressing sources of potential bias, imprecision, and, if relevant, multiplicity of analyses

20-i) Typical limitations in ehealth trials

Typical limitations in ehealth trials: Participants in ehealth trials are rarely blinded. Ehealth trials often look at a multiplicity of outcomes, increasing risk for a Type I error. Discuss biases due to non-use of the intervention/usability issues, biases through informed consent procedures, unexpected events.

	1	2	3	4	5	
subitem not at all important	○	○	○	○	◉	essential

Clear selection

Does your paper address subitem 20-i? *

Copy and paste relevant sections from the manuscript (include quotes in quotation marks "like this" to indicate direct quotes from your manuscript), or elaborate on this item by providing additional information not in the ms, or briefly explain why the item is not applicable/relevant for your study

"Limitations
This study has several limitations. First, it included only undergraduate nursing and physiotherapy students, and thus may not have captured the full range of perspectives and dynamics present in real-world interprofessional teams. The exclusion of those from other disciplines, such as medicine, pharmacy, social work, occupational therapy, and speech-language pathology, may have limited insights into the broader dynamics of collaboration, clinical decision-making, medication management, psychosocial support, and specialized rehabilitation. Second, most outcomes were assessed using self-reported questionnaires, which may have introduced an expectation bias; however, anonymity was implemented to reduce this bias. Additionally, qualitative findings cannot be generalized to a broader population since they are often based on personal experiences rather than on quantifiable data. Third, technical issues, such as difficulties with VR controls and connectivity, likely disrupted learning continuity and negatively affected student perceptions. These disruptions may have impacted immersion and collaboration, confounding assessments of the true educational impacts. Lastly, both IVR and web-based platforms lacked integrated patient interactions within simulation scenarios, which may have reduced the realism of the learning experience. Future studies should involve participants from a broader range of healthcare disciplines to enhance ecological validity, strengthen technical support and user training, and incorporate simulated patient encounters to improve authenticity, clinical reasoning, and engagement. Addressing these limitations will advance the development of technology-enhanced IPE and better prepare students for collaborative, real-world healthcare practice[68, 69]. "

21) Generalisability (external validity, applicability) of the trial findings

NPT: External validity of the trial findings according to the intervention, comparators, patients, and care providers or centers involved in the trial

21-i) Generalizability to other populations

Generalizability to other populations: In particular, discuss generalizability to a general Internet population, outside of a RCT setting, and general patient population, including applicability of the study results for other organizations

	1	2	3	4	5	
subitem not at all important	○	○	○	●	○	essential

Clear selection

Does your paper address subitem 21-i?

Copy and paste relevant sections from the manuscript (include quotes in quotation marks "like this" to indicate direct quotes from your manuscript), or elaborate on this item by providing additional information not in the ms, or briefly explain why the item is not applicable/relevant for your study

"In real-world practice, these findings suggest that educational institutions can strategically integrate a variety of serious game elements into IPE training to better prepare students for collaborative, patient-centered care."

21-ii) Discuss if there were elements in the RCT that would be different in a routine application setting

Discuss if there were elements in the RCT that would be different in a routine application setting (e.g., prompts/reminders, more human involvement, training sessions or other co-interventions) and what impact the omission of these elements could have on use, adoption, or outcomes if the intervention is applied outside of a RCT setting.

	1	2	3	4	5	
subitem not at all important	○	○	○	○	○	essential

Does your paper address subitem 21-ii?

Copy and paste relevant sections from the manuscript (include quotes in quotation marks "like this" to indicate direct quotes from your manuscript), or elaborate on this item by providing additional information not in the ms, or briefly explain why the item is not applicable/relevant for your study

Your answer

OTHER INFORMATION

23) Registration number and name of trial registry

Does your paper address CONSORT subitem 23? *

Copy and paste relevant sections from the manuscript (include quotes in quotation marks "like this" to indicate direct quotes from your manuscript), or elaborate on this item by providing additional information not in the ms, or briefly explain why the item is not applicable/relevant for your study

"In line with these guidelines, the study was retrospectively registered at ClinicalTrials.gov on 27 February 2026 (NCT07474701; https://clinicaltrials.gov/study/NCT07474701).This was an educational randomized study conducted among undergraduate healthcare students, with outcomes focused on training-related measures that included knowledge, attitudes, skills, and learning outcomes rather than patient health outcomes. Given the low-risk, non-clinical nature of the study and outcomes, the trial was not prospectively registered. "

24) Where the full trial protocol can be accessed, if available

Does your paper address CONSORT subitem 24? *

Cite a Multimedia Appendix, other reference, or copy and paste relevant sections from the manuscript (include quotes in quotation marks "like this" to indicate direct quotes from your manuscript), or elaborate on this item by providing additional information not in the ms, or briefly explain why the item is not applicable/relevant for your study

Full protocol can be accessed through the URL. "In line with these guidelines, the study was retrospectively registered at ClinicalTrials.gov on 27 February 2026 (NCT07474701; https://clinicaltrials.gov/study/NCT07474701)."

25) Sources of funding and other support (such as supply of drugs), role of funders

Does your paper address CONSORT subitem 25? *

Copy and paste relevant sections from the manuscript (include quotes in quotation marks "like this" to indicate direct quotes from your manuscript), or elaborate on this item by providing additional information not in the ms, or briefly explain why the item is not applicable/relevant for your study

"Declaration on the role of the funding body
The funder had no involvement in the study design, data collection, analysis, interpretation, or writing of the manuscript."

X27) Conflicts of Interest (not a CONSORT item)

X27-i) State the relation of the study team towards the system being evaluated

In addition to the usual declaration of interests (financial or otherwise), also state the relation of the study team towards the system being evaluated, i.e., state if the authors/evaluators are distinct from or identical with the developers/sponsors of the intervention.

	1	2	3	4	5	
subitem not at all important	○	○	○	○	◉	essential

Clear selection

Does your paper address subitem X27-i?

Copy and paste relevant sections from the manuscript (include quotes in quotation marks "like this" to indicate direct quotes from your manuscript), or elaborate on this item by providing additional information not in the ms, or briefly explain why the item is not applicable/relevant for your study

"Conflicts of Interest
The authors declare no conflicts of interest."

About the CONSORT EHEALTH checklist

As a result of using this checklist, did you make changes in your manuscript? *

- ○ yes, major changes
- ◉ yes, minor changes
- ○ no

What were the most important changes you made as a result of using this checklist?

Your answer

How much time did you spend on going through the checklist INCLUDING making changes in your manuscript *

3.5 hours

As a result of using this checklist, do you think your manuscript has improved? *

- (x) yes
- ( ) no
- ( ) Other:

Would you like to become involved in the CONSORT EHEALTH group?

This would involve for example becoming involved in participating in a workshop and writing an "Explanation and Elaboration" document

- ( ) yes
- ( ) no
- ( ) Other:

Any other comments or questions on CONSORT EHEALTH

Your answer

STOP - Save this form as PDF before you click submit
To generate a record that you filled in this form, we recommend to generate a PDF of this page (on a Mac, simply select "print" and then select "print as PDF") before you submit it.

When you submit your (revised) paper to JMIR, please upload the PDF as supplementary file.

Don't worry if some text in the textboxes is cut off, as we still have the complete information in our database. Thank you!

Final step: Click submit !
Click submit so we have your answers in our database!

Submit

Clear form

Never submit passwords through Google Forms.

This content is neither created nor endorsed by Google. - Contact form owner - Terms of Service - Privacy Policy

Does this form look suspicious? Report

Google Forms
